# Useful Ultrasonographic Parameters to Predict Difficult Laryngoscopy and Difficult Tracheal Intubation—A Systematic Review and Meta-Analysis

**DOI:** 10.3389/fmed.2021.671658

**Published:** 2021-05-28

**Authors:** Sara H. Gomes, Ana M. Simões, Andreia M. Nunes, Marta V. Pereira, Wendy H. Teoh, Patrício S. Costa, Michael S. Kristensen, Pedro M. Teixeira, José Miguel Pêgo

**Affiliations:** ^1^School of Medicine, Life and Health Sciences Research Institute (ICVS), University of Minho, Braga, Portugal; ^2^ICVS/3B's - PT Government Associate Laboratory, Braga, Portugal; ^3^Private Anesthesia Practice, Singapore, Singapore; ^4^Department of Anesthesia, Rigshospitalet, Copenhagen University Hospital, Copenhagen, Denmark

**Keywords:** airway ultrasound assessment, prediction of difficult intubation, prediction of difficult laryngoscopy, ultrasound predictors of difficult intubation, ultrasound predictors of difficult laryngoscopy

## Abstract

Unexpected difficult airway management can cause significant morbidity and mortality in patients admitted for elective procedures. Ultrasonography is a promising tool for perioperative airway assessment, nevertheless it is still unclear which sonographic parameters are useful predictors of difficult laryngoscopy and tracheal intubation. To determine the ultrasonographic predictors of a difficult airway that could be applied for routine practice, a systematic review and meta-analysis was conducted. Literature search was performed on PubMED, Web of Science and Embase using the selected keywords. Human primary studies, published in English with the use of ultrasonography to prediction of difficult laryngoscopy or tracheal intubation were included. A total of 19 articles (4,570 patients) were analyzed for the systematic review and 12 articles (1,141 patients) for the meta-analysis. Standardized mean differences between easy and difficult laryngoscopy groups were calculated and the parameter effect size quantified. A PRISMA methodology was used and the critical appraisal tool from Joanna Briggs Institute was applied. Twenty-six sonographic parameters were studied. The overall effect of the distance from skin to hyoid bone (*p* = 0.02); skin to epiglottis (*p* = 0.02); skin to the anterior commissure of vocal cords (*p* = 0.02), pre-epiglottis space to distance between epiglottis and midpoint between vocal cords (*p* = 0.01), hyomental distance in neutral (*p* < 0.0001), and extended (*p* = 0.0002) positions and ratio of hyomental distance in neutral to extended (*p* = 0.001) was significant. This study shows that hyomental distance in the neutral position is the most reliable parameter for pre-operative airway ultrasound assessment. The main limitations of the study are the small sample size, heterogeneity of studies, and absence of a standardized ultrasonographic evaluation method [Registered at International prospective register of systematic reviews (PROSPERO): number 167931].

## Introduction

### Rationale

Airway management is a core component of anesthesia care (1). In any procedure that requires general anesthesia, anesthesiologists need to control the patient's airway in order to maintain adequate ventilation and oxygenation. This can be a high-risk task and lead to patient morbidity and mortality, due to inadequate/impossible ventilation, and/or intubation. Therefore, it is essential to optimize methods to anticipate a difficult airway and ensure the necessary means to intervene (1).

According to the *Practice Guidelines for Management of the Difficult Airway* by the American Society of Anesthesiologists (ASA), a difficult airway is present when “a conventionally trained anesthesiologist experiences difficulty with facemask ventilation of the upper airway (…) tracheal intubation or both,” a laryngoscopy is difficult when “it is not possible to visualize any portion of the vocal cords after multiple attempts at conventional laryngoscopy” and an intubation is difficult when it “requires multiple attempts” (2). The etiology of a difficult airway is multifactorial and should prompt a detailed clinical history and physical examination (2–7).

However, most clinical predictors have low sensitivity and moderate specificity. Difficult/failed intubation has a low prevalence in the general population, and hence the positive predicted values (PPV) are also low (8). Even though there are several multivariate scoring systems which increase PPV in comparison to single tests, prediction scores still remain poor and many failures are still unanticipated as all airway management techniques can fail (4, 9).

Ultrasonography (US) is a promising tool for airway assessment, as it is safe, quick, repeatable, portable, widely available, and gives real-time dynamic images (10, 11). Many studies have recently been published in this field, but it is still unclear which sonographic parameters and respective cutoff values are clinically useful predictors of difficult laryngoscopy and intubation (11, 12).

### Objectives

This systematic review and meta-analysis was undertaken to identify and synthesize evidence from the existing literature (i) to determine the ultrasonographic predictors of difficult laryngoscopy and difficult tracheal intubation in anesthetized adult patients undergoing elective surgery, and to (ii) summarize the current knowledge and applicability of the sonographic measurements already trialed, in the hopes of contributing to establishing an ultrasonography standardized protocol for preoperative airway assessment.

## Methods

### Registration

The present review and meta-analysis was elaborated according to the transparent reporting of systematic reviews and meta-analyses, PRISMA (13, 14) and the study was registered at **International prospective register of systematic reviews** (PROSPERO): number 167931.

### Eligibility Criteria

The articles were considered when they fulfilled the following inclusion criteria: (1) Use of ultrasonography; (2) Prediction of difficult laryngoscopy or tracheal intubation; (3) Humans; (4) Primary studies; (5) English language. No time period was established, so all articles were included until search dates (12/04/2019 and 12/06/2019).

The exclusion criteria were: (1) Obstetric specialty; (2) Pediatric population; (3) Emergency context; (4) Laryngeal mask ventilation; (5) Gray literature. Reviews, editorials, conference abstracts and case reports were also excluded.

### Information Sources and Search Methods

The primary search was conducted using the following databases: PubMed, Web of Science and Embase. Keywords and Boolean operators used were: (ultrasound OR ultrasonic OR ultrasonography OR ultrasonographical OR sonography OR ultrasonographic) AND (predict OR predictor OR predictors OR prediction) AND (intubation OR laryngoscopy OR “airway management”) AND difficult. The search results were organized using a Microsoft Excel datasheet with records of the exclusion rationale and duplicated citations.

### Study Selection

**Figure 1:**
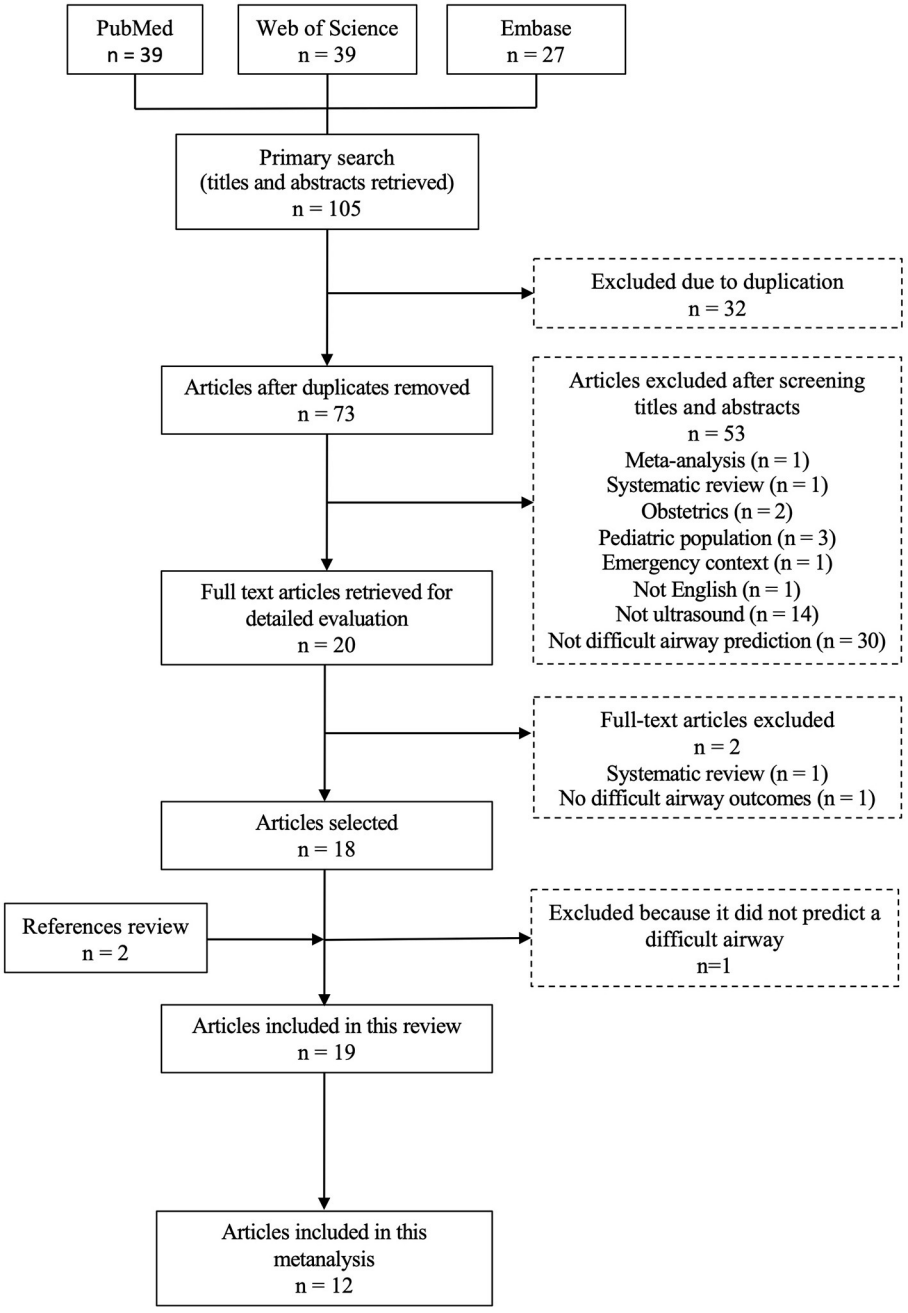
PRISMA methodology flowchart for article selection.

Three independent reviewers (AMS, AMN, MVP) screened studies on the basis of title and abstract to identify duplicates. The next step for screening was done by the same reviewers analyzing by title and abstract for eligibility and dissimilarities were solved by consensus after a review of full text publication by SHG. A full text analysis of the remain articles, for detailed information, was done by (AMS, AMN, MVP, SHG) and lead to article exclusion. The percent concordance value was calculated. In order to reduce the risk of publication bias, a reference list search of included studies and previous systematic review on the same topic was done (Figure 1).

### Data Collection Process

AMS, AMN, MVP extracted the data from each study and collected in a word file locally developed (similar to Cochrane Consumers and Communication review Group's data extraction template). SHG reviewed each data and supervised the process. Disagreements were resolved by discussion between authors. We contacted two authors for further information. One author responded and provided relevant information. No double counting was found. This study includes only two articles from the same author, but a different ultrasound parameter was studied in each article.

### Data Items

From each study, information was extensively extracted and included: (1) study design and methods (objective, study design, anesthesiologist blinding process, standard characteristics—age, sex, BMI, clinical evaluation, US measurements, position for US measurements and laryngoscopy technique); (2) sample selection (number of participants, BMI, demographic characteristics, inclusion criteria, and setting—Hospital and/or country); (3) Exclusion criteria; (4) Variables and data type (dependent and independent); (5) Statistical analysis (data normality test, numerical, ordinal, nominal, correlation, regression, ROC curve, …); (6) US results; (7) Conclusions and (8) Limitations. The data was extracted and organized in Supplementary Table 1 and the relevant cutoff values, means and standard deviations from the predictors were gathered in Supplementary Table 2. To standardize the presentation of results, all distance measurements were converted into centimeters.

### Risk of Bias in Individual Studies

The quality assessment of each study was done using the “Checklist for Analytical Cross Sectional Studies” tool, by *The Joanna Briggs Institute* (JBI), by four independent reviewers (AMS, AMN, MVP, SHG) (15) (**Appendix 1**).

### Summary Measures and Planned Methods of Analysis

The analysis of the obtained data is presented in forest plots developed using Review Manager 5.3 software (16).

Considering the heterogeneity of the studies, random effect modeling was chosen, and the effect size measured by Standardized Mean Difference to allow comparison of the results. Authors tested heterogeneity using the method proposed by Higgins at al. (17) to measure inconsistency (*I*^2^). An value of *I*^2^ superior to 50% was considered significant which indicated a lower reliability of results.

In order to enable comparison with the other results, a study that presented results as mean and confidence interval, the standard deviation was calculated using the following formula: (SQRT(n)x(Upper CI- Lower CI)/t_α,df_ x2) being the mean and n values relative to the difficult group of each specific parameter (18).

To allow the comparison with other studies, the 4 categories of Cormack-Lehane classification, was dichotomized as easy and difficult by calculating the weighted average of the mean and standard deviation for each group (grades 1 and 2 vs. grades 3 and 4) (19–21).

### Ethics

Ethical approval for this study was not required because no animals or patients were involved.

## Results

### Study Selection

The flowchart in Figure 1 describes the search method implemented, following PRISMA statement. Primary search on PubMed and Web of Science occurred at 12/04/2019 and Embase at 12/06/2019. PubMed's search was sorted by the “most recent” results and filtered by humans, English and adults (≥18 years), obtaining 39 articles. Web of Science's search was sorted by “topic” and filtered by articles and English, excluding the following filters: obstetrics and gynecology, and pediatrics, obtaining 39 articles. The following filters were used in Embase's search: young adult, adult, middle aged, aged, very elderly and articles, and the results were sorted by “all fields,” obtaining 27 articles.

One hundred and five articles were identified through database search. Thirty two duplicates were identified. Seventy three studies were analyzed by title and abstract by reviewers for eligibility and dissimilarities were solved by consensus after a review of full text publication by SHG. Due to the particular inclusion/exclusion criteria of this study, this step of screening was possible to do only with title and abstracts analysis, without significant bias, and 53 articles were excluded (see reasons in Figure 1). After a full text analysis of 20 articles, for detailed information, two papers were excluded, with a percent concordance value of 1. In order to reduce the risk of publication bias, we search reference list in included studies and previous systematic review on the same topic and one more article was included. Therefore, 19 articles were included in the systematic review and 12 in the meta-analysis (Figure 1).

### Studies Characteristics

This review included 4,570 adult patients undergoing elective surgery with general anesthesia and tracheal intubation. A summary of the main characteristics, conclusions, and limitations of each article included in the present systematic review and meta-analysis is presented in Supplementary Table 1.

All studies analyzed were cross-sectional observational studies. Most of them were prospective (94.7%), except Wojtczak's (12) study where previous anesthesia records were reviewed (5.3%). Blinding was assured in 73.6% of studies, not mentioned in 21.1% (12, 21–23) and “not guaranteed” in 5.3% (24).

The most common exclusion criteria reported were limited neck mobility secondary to cervical spine fractures or tumors (18–22, 24–30), limited neck extention (21, 22, 29, 31, 32), including arthritis (24). Patients with maxillofacial fractures or deformities (18, 21, 22, 24, 27–29, 31, 32) or upper airway abnormalities (19, 25, 26, 28, 30, 33–35), including epiglottic surgery (24), limited interincisal distance (20, 22, 29, 31, 32), subglottic stenosis (35), or thyroid disease (27, 30) were also eliminated from the reported samples.

Although upper teeth removal or absence (20–22, 24, 26) can improve the interincisal distance and facilitate the introduction of the laryngoscope and the direct laryngoscopy view, only (20–22, 24, 26) excluded these patients from their studies.

Previous history of difficult intubation (25, 26, 28, 32, 33, 35) and pregnancy (19, 25–27, 30, 32) were disqualifiers in 32% of the studies.

Patients with full stomach (21, 22), diaphragmatic hiatus hernia (21, 22), gastroesophageal reflux disease (GERD) (25, 26), and planned rapid sequence intubation (19, 23, 32) were excluded in some studies. Only Wojtczak (12) didn't mention exclusion criteria.

Ninety percent of studies reported standard characteristics of age, sex, body mass index; with Reddy et al. (19) only considering the body mass index and no mention of this parameter in Gupta et al. (21) study. Two studies recorded the race of the patients (15, 34).

A population of obese patients were specifically studied in 21.1% of the articles (12, 23, 25, 26). Pinto et al. (27), Mohammadi Soltani (22), Parameswari's et al. (31), and Rana et al. (20) excluded this population from their studies, however in Reddy's study (19) a population with a heterogenous weight was included.

Difficult airway, clinically relevant history and objective signs from the physical examination were collected by the majority, except for Falcetta et al. (32) who did not mention which clinical screening evaluation was used.

A history of Obstructive Sleep Apnea Syndrome (OSAS) was collected in 21.1% of the studies (18, 25, 26, 30); teeth patology (18, 25, 26, 30, 34) and neck mobility problems (18, 25, 26, 30, 34) in 26%, and neck circumference (12, 18, 25–27, 31) in 32% of the studies.

The mobility of the temporomandibular joint was directly evaluated in only 10.5% of studies (18, 26), though many other authors evaluated parameters related to that joint's kinesis, for example interincisal distance (IID) (18, 20, 24–28, 30, 31, 33–35), upper lip bite test (ULBT) (23, 28, 30, 33), mandibular protusion (28), and condyle-tragus distance (28).

The evaluation of the clinical predictors was done by the same practitioner in a minority of the studies (19, 25, 26), with only (28, 33, 35) stating that the evaluation was done by clinicians with competency for the task.

In 53% of the studies (19, 20, 23, 24, 28, 30, 32–35), laryngoscopy was reportedly performed by experienced anesthetists with more than 2 years of training, although most of the authors (53%) omitted mentioning the number of anesthetists who performed the technique (12, 19, 20, 22, 23, 27, 29, 31, 32, 35).

In all studies a direct laryngoscopy was used and the Cormack-Lehane (CL) grade was defined as the outcome variable (CL 1&2 = easy vs. CL 3&4 = difficult). In order to facilitate the laryngoscopy view, a backward, upward, and rightward pressure (BURP) was applied on the thyroid cartilage during all laryngoscopies in Ezri et al. (25) study. In Komatsu et al. (26), Yao and Wang (35), and Falcetta et al. (32) studies this maneuver was also allowed, however Prestrisor et al. (23) excluded patients when an external laryngeal manipulation was necessary.

Ultrasound measurements were done by the same sonographer in 58% of the studies (18, 21, 24, 24–28, 30, 31, 34), and in 42% the experience of the ultrasound practitioner was not declared (12, 19, 20, 22–24, 27, 30).

Another essential aspect of the studies is the positioning of the patient in which US parameters were evaluated. All US measurements were conducted in supine position, except in Hui and Tsui (34) and Yao et al. (28) studies (sitting position) (10.5%) and Ezri et al. (25) did not specify this feature (5.3%).

The majority of the measurements of the skin to structure distance were undertaken in the central axis of the neck, but Erzi et al. (25), Komatsu et al. (26), Adhikari et al. (18), Pinto et al. (27), and Falcetta et al. (32) presented averaged values from the measurements taken in the central midline and 1.0 or 1.5 cm to each side.

### Risk of Bias Within Studies

Quality assessment of the studies included in the systematic review and the meta-analysis was done using the “Checklist for Analytical Cross Sectional Studies” tool, by *The Joanna Briggs Institute* (JBI), by four independent reviewers (15) (**Appendix 1**).

All studies have clearly defined the criteria for sample selection except for Wojtczak (12) that does not mention exclusion criteria. Ezri et al. (25), Komatsu et al. (26), Gupta et al. (21), Parameswari et al. (31), Wojtczak (12), Rana et al. (20), and Petrisor et al. (23) did not describe in detail the demographics, location, or time period of their studies; whereas, Wu et al. (24), Pinto et al. (27), Andruszkiewicz et al. (33), Reddy et al. (19), Yao et al. (28), and Chan et al. (29) left some of these parameters unclear.

The description of the study subjects, setting, and time was presented in detail in Adhikari et al. (18), Andruszkiewicz et al. (33), Yao et al. (28), Yao and Wang (35), Falcetta et al. (32), Mohammadi Soltani et al. (22), and Alessandri et al. (30).

In relation to the validity of the studies performing US evaluations, almost every study described in detail the technique implemented to obtain the measurements, with the exception of three (25, 26, 34). Furthermore, Yao et al. (28) and Yao and Wang (35) studies assessed inter-rater reliability by comparing the measurements of at least two independent sonographers, and (29) assessed both inter and intra-rater reliability.

All studies selected only patients undergoing elective surgery, therefore standard criteria were used to evaluate the referred condition. Although none of the studies has listed eventual confounding factors, (12, 23, 25, 26) selected only obese patients; (20, 32) excluded patients with clinically predicted difficult airway; (19, 21, 22, 24, 30) performed a correlation analysis between the considered variables and (26, 28, 33, 35) studies performed a multivariate logistic regression analysis to assess the potential effect of each variable while controlling the effect of others.

Regarding the laryngoscopy classification method, there are some important differences between studies since Chan et al. (29) analyzed anesthesia records after surgeries (12), analyzed anesthesia records from previous surgeries, and (18, 21, 22, 28) did not have an anaesthesiologist specifically assigned for this task. Furthermore, Wu et al. (24) did not blind the US results and (12, 21–23) did not mention blinding.

With regards to statistical analysis, the majority of studies used appropriate methods. However, not all results were reported in Adhikari et al. (18), Parameswari et al. (31), Mohammadi Soltani et al. (22), and Falcetta et al. (32) studies. Some presented unclear statistical data, such as Gupta et al. (21), Rana et al. (20), Parameswari et al. (31), and Petrisor et al. (23). Finally, Ezri et al. (25), Komatsu et al. (26), Wu et al. (24), Pinto et al. (27), Mohammadi Soltani et al. (22), Wojtczak's (12), Yao et al. (28), and Yao and Wang (35) studies did not mention the application of normality tests, which may compromise the results, especially in Wojtczak's (12) study, due to the small sample size.

### Results of Studies by Ultrasound Parameter

To predict difficult laryngoscopy and difficult tracheal intubation, a total of 26 US parameters were investigated in the 19 studies.

#### Significant Ultrasound Predictors of Difficult Laryngoscopy

The following parameters were significant in predicting a *difficult laryngoscopy*: evaluation of the distance from skin to hyoid bone (24), skin to epiglottis (18, 23, 24, 27, 32), skin to vocal cords (VC) (19, 24–26), and skin to anterior aspect of trachea at the level of suprasternal notch (25); condylar translation (28); HMD in neutral (23, 33), ramped (23), and extended (27, 31) position; tongue cross-sectional area and volume (33), thickness and ratio of tongue thickness to TMD (35); Pre-E/aVC (29); Pre-E/mVC (20, 21); ratio between HMD ramped position and neutral position (HMDR1) (23); ratio between HMD in the extended position and neutral position (HMDR2) (12, 20, 23, 33); pre-epiglottic area (PEA) (32) and visualization of hyoid bone with sublingual US (34) approach.

By contrast, evaluation of the pre-epiglottic space (Pre-E) (22), distance from epiglottis to midpoint of the distance between vocal cords (E-VC) (22); skin to trachea at the level of the thyroid isthmus (18, 25, 30); floor of the mouth muscle cross-sectional area (33); floor of the mouth muscle volume (12, 27); tongue width and tongue thickness-to-oral cavity height ratio (33) and Pre-E/pVC (22, 29) were not significant in predicting a difficult laryngoscopy.

Outcomes from each study was profoundly analyzed, we present the most relevant.

##### Hyoid Bone Visualization

Hui et al. (34) study concluded that sublingual ultrasound had a sensitivity of 73% and a specificity of 97% for predicting difficult intubation (*p* < 0.0001) when hyoid bone visualization was not possible.

##### Skin to Hyoid Bone

**Figure 2:**
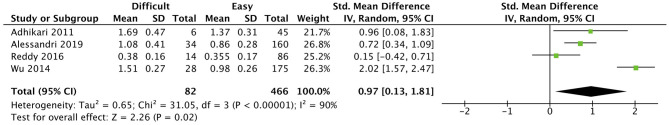
Skin to Hyoid bone distance forest plot comparing difficult and easy laryngoscopy groups.

At the level of the hyoid bone, patients with a difficult laryngoscopy had a significantly larger distance from skin to hyoid bone of 1.08 ± 0.41 cm (30), 1.69 ± 0.62 cm (18), and 1.51 ± 0.27 cm (24) compared with easy laryngoscopy. Wu et al. (24) concluded that a distance more than 1.28 cm predicts a difficult laryngoscopy (Se: 85.7%, Sp: 85.1%). By contrast, findings in Reddy et al. (19) study were not statistically significant. The overall effect of this measurement was significant (*p* = 0.02) (Figure 2).

##### Skin to Epiglottis

**Figure 3:**
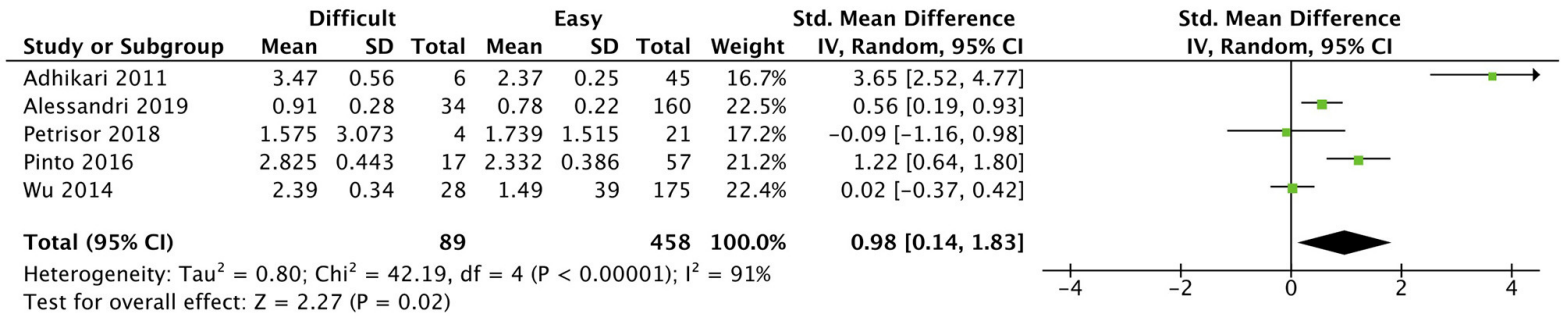
Skin to Epiglottis distance at THM level forest plot comparing difficult and easy laryngoscopy groups.

At the level of the thyrohyoid membrane, patients with a difficult laryngoscopy displayed mean measurements over 2.8 cm (18), 1.78 cm (24) (Se: 100%, Sp: 66.2%), 2.54 cm (32) (Se: 82%, Sp: 91%), and equal or superior to 2.75 cm (27) (Se: 64.7%, Sp: 77.1%). Even a mere 0.91 ± 0.28 cm (30) was found to be associated with difficult laryngoscopy. By contrast, findings in Petrisor et al. (23) study were not statistically significant. The overall effect of this measurement was significant (*p* = 0.02) (Figure 3).

##### Skin to Vocal Cords

**Figure 4:**
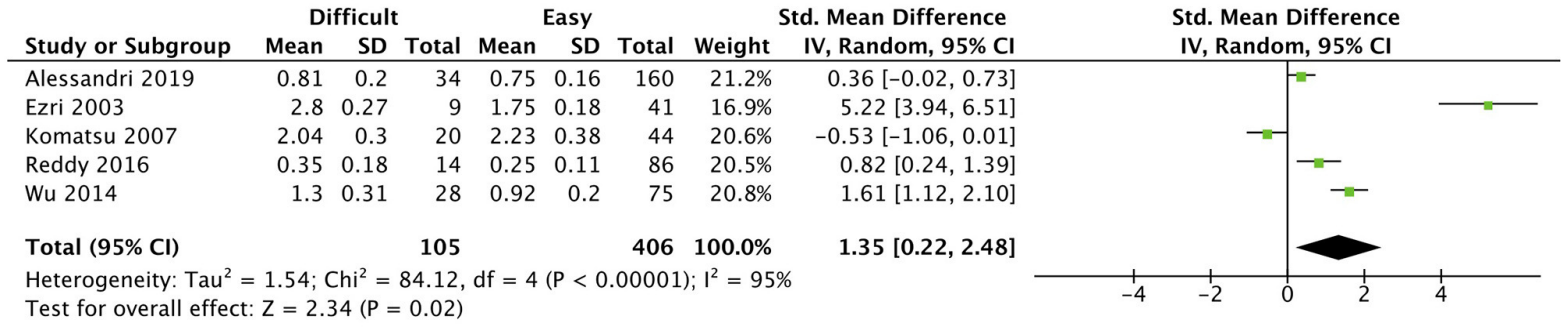
Skin to anterior commissure of vocal cords distance forest plot comparing difficult and easy laryngoscopy groups.

At the level of the vocal cords, studies showed significant discrepancies. While Ezri et al. (25) reported that patients with a difficult laryngoscopy presented a significantly bigger distance from skin to vocal cords (2.80 ± 0.27 cm compared to easy laryngoscopy, 1.75 ± 0.18 cm), Komatsu et al. (26) reported an inverse relationship between difficult and easy laryngoscopy patients (2.04 ± 0.3 and 2.23 ± 0.38 cm, respectively). A distance superior to 1.10 cm (Se: 75%, Sp:80.6%) (24) and even 0.23 cm (Se: 85.7%, Sp: 57%) (19) predicted a difficult laryngoscopy in other studies. By contrast, findings in Adhikari et al. (18), Falcetta et al. (32), and Alessandri et al. (30) studies were not statistically significant. However, the overall effect of this measurement was significant (*p* = 0.02) (Figure 4).

##### Pre-epiglottic Area (PEA)

The pre-epiglottic area analyzed by Falcetta et al. (32) measures the area from skin to epiglottis 10 mm each side of the midline should be not confused with the pre-epiglottic distance used by Gupta et al. (21), Chan et al. (29), Rana et al. (20), or Mohammadi et al. (22) (see definition above). Falcetta et al. (32) concluded that if this measurement was superior to 5.04 cm^2^ (Se: 85%, Sp: 88%), it predicted a difficult laryngoscopy.

##### Pre-epiglottic Space (Pre-E) and Distance From Epiglottis to Midpoint Between Vocal Cords (E-VC)

Only Mohammadi Soltani (22) evaluated Pre-E and E-VC in isolation and concluded that the correlation between Pre-E and E-VC with Cormark-Lehane grade 1–3 were weak.

##### Pre-epiglottic Space to Distance Between Epiglottis and Midpoint Between the Anterior and Posterior Vocal Cords Ratio (Pre-E/mVC)

**Figure 5:**
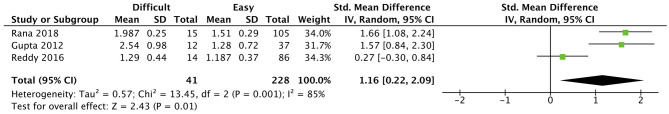
Ratio between the pre-epiglottic space depth and the distance from epiglottis to the midpoint of the vocal cords (Pre-E/mVC) forest plot comparing difficult and easy laryngoscopy groups.

Rana et al. (20) established that a Pre-E/mVC ratio superior to 1.77 (Se: 82%, Sp: 80%) predicts a difficult laryngoscopy and (21) described a strong positive correlation with a regression coefficient of 0.495 (95% CI 0.319–0.671; *p* < 0.0001) even though (19) did not obtain a statistically significant result for this parameter. The overall effect was significant (*p* = 0.01) (Figure 5).

##### Pre-epiglottic Space to Distance Between Epiglottis and Anterior Vocal Cord Ratio (Pre-E/aVC)

Chan et al. (29) found that a Pre-E/aVC ratio superior to 1 (Se: 79.5%; Sp: 39.2%) (29) predicts a difficult laryngoscopy.

##### Skin to Anterior Aspect of Trachea at the Level of Thyroid Isthmus

**Figure 6:**
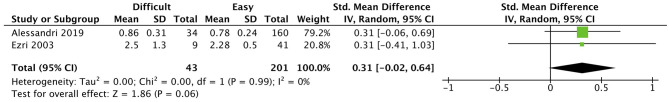
Skin to anterior aspect of the trachea distance at the level of thyroid isthmus forest plot comparing difficult and easy laryngoscopy groups.

This was not statistically significant in the individual studies (18, 25, 30) that considered this measurement. Its overall effect was also not significant (*p* = 0.06) (Figure 6).

##### Skin to Anterior Aspect of Trachea at the Level of Suprasternal Notch

**Figure 7:**
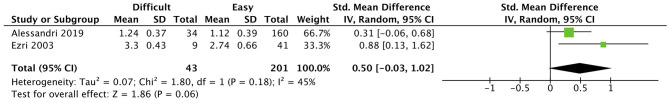
Skin to anterior aspect of the trachea distance at the level of suprasternal notch forest plot comparing difficult and easy laryngoscopy groups.

At the suprasternal notch, patients with a difficult laryngoscopy had significantly deeper skin to the anterior aspect of trachea distance of 3.30 ± 0.43 cm (25). By contrast, findings in Adhikari et al. (18) and Alessandri et al. (30) studies were not statistically significant. The overall effect of this measurement was also not significant (*p* = 0.06) (Figure 7).

##### Hyomental Distance in Neutral Position

**Figure 8:**
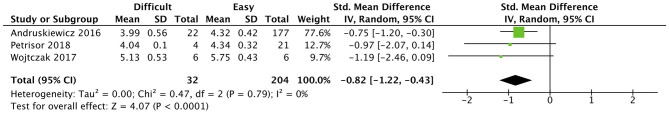
HMD in neutral position forest plot comparing difficult and easy laryngoscopy groups.

Patients with shorter hyomental distances in neutral position [3.99 ± 0.56 cm (33)] were found to be significantly associated with difficult laryngoscopy. Although Petrisor et al. (23) and Wojtczak (12) did not obtain statistically significant results for this parameter, the overall effect of this measurement was significant nonetheless (*p* < 0.0001) (Figure 8).

##### Hyomental Distance in Ramped Position

Petrisor et al. (23) concluded that an HMD in ramped position equal or inferior to 4.97 cm (Se: 100%, Sp: 61.9%) predicts a difficult laryngoscopy.

##### Hyomental Distance in Extended Position

**Figure 9:**
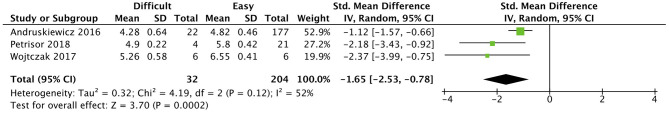
HMD in extended position forest plot comparing difficult and easy laryngoscopy groups.

Patients with a difficult laryngoscopy had significantly decreased HMD in the extended position, of 4.28 ± 0.64 cm (33) and 5.26 ± 0.58 cm (12), compared to patients with easy laryngoscopy. Petrisor et al. (23) established that values equal or inferior to 5.50 cm (Se: 100%, Sp: 71.4%), predicted a difficult laryngoscopy. The overall effect of this measurement was significant (*p* = 0.0002) (Figure 9).

##### Hyomental Distance in Ramped to Neutral Position Ratio (HMDR1)

Petrisor et al. (23) found that HMDR1 equal or inferior to 1.12 (Se: 75%, Sp: 76.2%) predicts a difficult laryngoscopy.

##### Hyomental Distance in Extended to Neutral Position (HMDR2)

**Figure 10:**
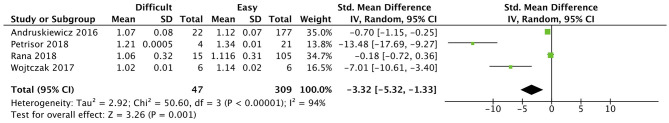
Ratio between HMD in extended position and HMD in neutral (HMDR2) forest plot comparing difficult and easy laryngoscopy groups.

Patients with a difficult laryngoscopy presented with a significantly shorter HMDR2 [1.07 ± 0.08 (33) and 1.02 ± 0.01 (12)] compared with patients with easy laryngoscopy. HMDR2 equal or inferior to 1.085 (Se: 75%, Sp: 85.3%) (20) and to 1.23 (Se: 100%, Sp: 90.5%) (23) predicted a difficult laryngoscopy. The overall effect of this measurement was significant (*p* = 0.001) (Figure 10).

##### Tongue Volume

**Figure 11:**
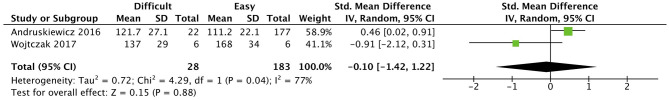
Tongue volume forest plot comparing difficult and easy laryngoscopy groups.

The group of difficult laryngoscopy patients in Andruskiewicz's study (33) had significantly larger tongue volumes (121.7 ± 27.1 cm) compared with patients with an easy laryngoscopy. Wojtczak (12) did not obtain a statistically significant result for this parameter. The overall effect of this measurement was also not significant (*p* = 0.88) (Figure 11).

##### Floor of the Mouth Muscle Volume

**Figure 12:**
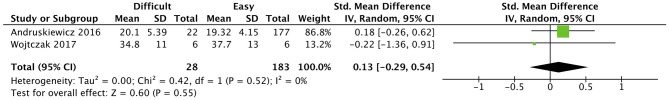
Floor of mouth muscle volume forest plot comparing difficult and easy laryngoscopy groups.

The floor of the mouth muscle volume parameter was not statistically significant in individual studies (12, 31); its overall effect was not significant as well (*p* = 0.55) (Figure 12).

##### Tongue Thickness

Yao and Wang (35) concluded that in difficult laryngoscopy patients had larger tongue thickness, a value superior to 6.0 cm (Se: 63%, Sp: 66%) predicts a difficult laryngoscopy. By contrast, in Adhikari et al. (15) study the parameter was not considered statistically significant, although the values were not presented.

##### Tongue Cross-Sectional Area

Only Andruszkiewicz et al. (33) evaluated tongue cross-sectional area and concluded that patients with a difficult laryngoscopy had a larger tongue cross sectional area (23.1 ± 3.57 cm^2^) compared with the easy laryngoscopy group (21.6 ± 3.09 cm^2^).

##### Condylar Translation

Only Yao et al. (28) evaluated condylar translation and concluded that if this measurement was equal or inferior to 1 cm [sensitivity (Se): 81%, specificity (Sp): 91%] it predicted a difficult laryngoscopy.

An overall view of the results of the difficult laryngoscopy group from the studies included in the meta-analyses is presented in Supplementary Table 2.

#### Significant Ultrasound Predictors of Difficult Tracheal Intubation

As predictors of difficult tracheal intubation, only three parameters were analyzed. Tongue thickness (35) was a ***significant*** predictor, whereas the distance from skin to anterior commissure of vocal cords (26) and tongue thickness to TMD ratio were not significant. Yao et al. (35) determined that a measurement of tongue thickness superior to 6.1 cm (Se: 75%, Sp: 72%) and tongue thickness to TMD ratio superior than 0.87 (Se: 84%, Sp: 79%) predicted a difficult tracheal intubation. On the other hand, Komatsu et al. (26) did not obtain a statistically significant result when analyzing the distance from skin to the anterior commissure of vocal cords. Therefore, Yao and Wang study (35) is the only one that obtained a statistically significant result for difficult tracheal intubation.

No forest plot was done for difficult tracheal intubation predictors since for each ultrasound parameter studied only one paper was published.

### Syntheses of Results

To predict a difficult laryngoscopy, 12 articles were analyzed and 11 ultrasound parameters were included in the meta-analysis, 7 of which had a significant overall effect.

Concerning the distance from skin to the hyoid bone, the positioning of patients may explain why (19) was the only study without significant results [head in extended (19) vs. neutral position (18, 24, 30)]; a sample of Asian population could have also contributed to this outcome. Even though the meta-analysis had a significant result, the high heterogeneity (*I*^2^ of 90%) means it is less reliably applied in clinical practice.

Adhikari et al. (18), Pinto et al. (27), Wu et al. (24), Alessandri et al. (30), and Petrisor et al. (23) studies analyzed the distance from skin to epiglottis and had different results from their studies.

The methodology adopted by Adhikari et al. (18) and Pinto et al. (27) were very similar. Both excluded morbidly obese patients and the ultrasound images were collected by the same sonographer with the same transducer (Sonosite M-Turbo, 10 MHz linear). The positioning of the patients was also comparable (neutral position without a pillow). The CL categorization was equal (CL 1&2 easy vs. CL 3&4 difficult). In Adhikari et al. (18) reported values that corresponded to the average of three measurements (one made in the central axis and two measurements distanced 1 cm from the central axis on either side) while Pinto et al. (27) reported similar methodology except that the lateral measurement was made at the lateral border of the epiglottis.

Eighty and 76% of the difficult airway patients were males and a cut-off of >2.8 and ≥2.75 cm was established in Adhikari et al. (18) and Pinto et al. (27), respectively. Although the methodology was quite similar to the studies referred above (i.e., transducer, patient positioning), Wu et al. (24) study selected patients from Chinese Han population, that were less heavy than in Pinto et al. (27). This fact can explain why its cut-off value of 1.78 cm (Se: 100%, Sp: 66.3%) is less than the studies mentioned above (>2.8 cm in Adhikari's and ≥2.75 cm in Pintos's study).

Skin to epiglottis distance showed significant differences between the difficult and the easy laryngoscopy groups in all studies except in Petrisor's study (23). The study population was morbidly obese (i.e., BMI>40 kg/m^2^), 75% of the difficult airway patients were female, and the patient position for collecting this measurement was omitted. Those facts can explain the differences from Adhikari et al. (18), Pinto et al. (27), Wu et al. (24), and Alessandri et al. (30). Although its significant value in meta-analysis, strong evidence of heterogeneity (*I*^2^ = 91%, *p* < 0.00001) was observed.

The distance from skin to the anterior commissure of the vocal cords studied by Ezri et al. (25), Komatsu et al. (26), Wu et al. (24), Reddy et al. (19), and Alessandri et al. (30) had a significant overall effect but a low reliability.

The study design, methods and sample selection were very similar in Ezri et al. (25) and Komatsu et al. (26) which can explain the similarity of results (2.8 ± 0.27 vs. 2.04 ± 0.3 cm). Although Wu et al. (24) adopted the same methodology and study design, their sample of lighter Chinese Han population may have resulted in a lower cut-off and lower mean (>1.1 cm, 1.30 ± 0.31 cm). Reddy et al. (19) yielded the most discrepant result (cutoff >0.23 cm) compared to the studies above, mostly secondary to a specific population (only 6% of patients were obese) and the extended cervical position adopted for the US evaluation. Alessandri et al. (30) was the only study without significant results and with the lowest mean value measured. This authors adopted a different CL categorization (CL grade 2B as difficult laryngoscopy), which may have unintentionally placed patients in the difficult laryngoscopy group, that in other studies would belong to the easy group. A strong heterogeneity was reported (*I*^2^ = 95%, *p* < 0.00001).

Rana et al. (20) and Gupta et al. (21) found significant results concerning Pre-E/mVC. In Gupta et al. (21) study the mean value for the Pre-E/E-VC was much higher (2.54 cm ± 0.98) when compared with Reddy et al. (19) (1.29 cm ± 0.44) and Rana et al. (20) (1.987 cm ± 0.26) results. This can be partially explained by a small sample size, an incomplete investigator training and by the unknown demographics from Gupta et al. (21) study. Rana et al. (20) included patients with a BMI <25 kg/m^2^ and Reddy (19) between 14.2 and 39 kg/m^2^, which may explain the distinct results and indicate that this parameter might be more useful in normal weighted patients. Although the overall effect was significant, the heterogeneity (*I*^2^ = 85%) was high.

Only Andruskiewicz et al. (33) found significant differences measuring HMD in neutral position. This parameter had, however, a significant overall effect, with 0% heterogeneity, since this author and co-workers had a considerably higher weight in the analysis due to the sample size (199 patients). This US parameter was the most reliable measure of a difficult laryngoscopy.

Both HMD in extended position (12, 23, 33) and HMDR2 (12, 20, 23, 33) had significant overall effects as all studies had significant differences. All authors implemented the same methodology, study design and US measurement technique, for both HMD extended and HMDR2. Although Petrisor et al. (23) and Wojtzak (12) studied obese and morbidly obese patients, the population body mass index in Andruszkiewicz et al. (33) study had the lowest BMI while in Rana et al. (20) patients had variable BMI (14.2–39 kg/m^2^). This fact suggests that this predictor may be applied to the general population. However, HMD in extended position had a heterogeneity of 52%, meaning that their relevance for clinical practice is still unclear.

## Discussion

### Summary of Evidence

Despite its widespread use, ultrasonography is not yet routinely used for airway assessment and management (36–39) and its use for prediction of a difficult airway is still limited.

In the literature, several ultrasound measurements of cervical anatomic structures have been assessed and used as indicators of difficult airway, but there is still debate about the best parameter and the need for higher level of evidence (40).

The present analysis revealed that 7 US measurements have a significant overall effect as predictors for a difficult laryngoscopy. We found that HMD in neutral position was the most consistent predictor. Other potentially useful measurements are HMD in extended position, HMDR2, Pre-E/E-mVC, as well as the distance from skin to hyoid bone, skin to epiglottis, and skin to the anterior commissure of vocal cords.

### Limitations

One of the most important limitations of this systematic review and meta-analysis is the heterogeneity of the samples in the studies reviewed, namely BMI ranges, ethnic diverse populations, and female to male proportions. There is also significant discrepancy in study characteristics, mainly in the sample size and blinding. Another limitation of the study is related to the concepts of difficult laryngoscopy and difficult intubation. Both words are used interchangeably in same cases. Therefore, the use of the Cormack-Lehane classification as a surrogate outcome measure for a difficult tracheal intubation may imply a bias in this study. However, it has been thoroughly utilized in the literature, and a CL grade 3 was associated with an 87.5% likelihood of a difficult tracheal intubation (41).

In the selected studies, there may be a selection bias as only articles related to elective surgeries were chosen, which can compromise external validity of the results. This resulted from the fact that only one article on the use of ultrasound in emergency surgery was identified and was not considered as representative sample. Additionally, our population study excluded pregnant (42, 43) and pediatric (44, 45) patients. Both groups of populations have airway anatomical and physiological specificities that could render a bias in the analysis and should be analyzed independently from the general population.

Finally, for the evaluation of each US parameter, a standardized US technique and positioning are missing, hence there may be a bias associated with the acquisition of the data, even though there was a significant effort to describe in detail the used technique and to train the sonographers to allow reproducibility of the results.

## Conclusion

Our findings suggest that ultrasonography is a useful tool for prediction of a difficult laryngoscopy and that the best candidate to implement in clinical practice is the measurement of the hyomental distance with the head and neck in neutral position.

### Recommendations for Future Studies

Future studies should include larger sample sizes with proportional standard characteristics and ensure a standardized US measurement technique and positioning. It would also be beneficial to assess inter and intra-rater reliability to ensure the validity of the results.

Assessing HMD in neutral position may be the direction to go as it is the most promising US parameter. It may be relevant to define a specific cut-off for ethnicity, obesity, pregnancy, and pediatric patients and implement US airway evaluation in the context of emergency and intensive care.

Finally, it would be advantageous to introduce ultrasonography for the preoperative airway assessment in anesthesiology curriculum training (46), to ensure the acquisition of the skill as early as possible.

## Data Availability Statement

The raw data supporting the conclusions of this article will be made available by the authors, without undue reservation.

## Author Contributions

SG and JP: study conception and design. SG, AS, AN, MP, PT, PC, and JP: acquisition of data, analysis and interpretation of data, and drafting the article. SG, WT, MK, PT, PC, and JP: in depth revising the manuscript critically for important intellectual content. SG, AS, AN, MP, WT, PC, MK, PT, and JP: final approval of the version to be published and all agree to be countable for all aspects of the work thereby ensuring that questions related to the accuracy or integrity of any part of the work are appropriately investigated and resolved. All authors contributed to the study substantially.

## Conflict of Interest

The authors declare that the research was conducted in the absence of any commercial or financial relationships that could be construed as a potential conflict of interest.

## References

[1] O'Dell K. Predictors of difficult intubation and the otolaryngology perioperative consult. Anesthesiol Clin. (2015) 33:279–90. 10.1016/j.anclin.2015.02.002 25999002

[2] Apfelbaum JL Hagberg CA Caplan RA Blitt CD Connis RT Nickinovich DG. Practice guidelines for management of the difficult airway: an updated report by the american society of anesthesiologists task force on management of the difficult airway. Anesthesiology. (2013) 118:251–70. 10.1097/ALN.0b013e31827773b2 23364566

[3] Bajracharya G Truong A Truong D Cata J. Ultrasound-assisted evaluation of the airway in clinical anesthesia practice: past, present and future. Int J Anesthesiol Pain Med. (2015) 1:1–10. http://anesthesia-painmedicine.imedpub.com/archive.php [accessed 12 June 2019]

[4] Crawley S Dalton A. Predicting the difficult airway. BJA Educat. (2015) 15:253–57. 10.1093/bjaed/mku047

[5] Shirgoska B Netkovski J. Predicting difficult airway in apparently normal adult and pediatric patients. Pril. (2013) 34:155–9. 23917749

[6] Krage R Van Rijn C Van Groeningen D Loer SA Schwarte LA Schober P. Cormack-Lehane classification revisited. Br J Anaesth. (2010) 105:220–7. 10.1093/bja/aeq136 20554633

[7] Cormack RS Lehane J. Difficult tracheal intubation in obstetrics. Anesthesia. (1986) 41:332–3. 10.1111/j.1365-2044.1986.tb12815.x 6507827

[8] Australian and New Zealand College of Anaesthetists. Airway Assessment – ANZCA (2016). Available onlin at: http://www.anzca.edu.au/documents/pu-airway-assessment-20160916v1.pdf (accessed at: 12 June 2019).

[9] Cook TM Macdougall-Davis SR. Complications and failure of airway management. Br J Anaesth. (2012) 109(suppl 1):i68–85. 10.1093/bja/aes393 23242753

[10] Kristensen MS. Ultrasonography in the management of the airway. Acta Anaesthesiol Scand. (2011) 55:1155–73. 10.1111/j.1399-6576.2011.02518.x 22092121

[11] Petrişor C Dîrzu D Trancă S Hagău N Bodolea C. Preoperative difficult airway prediction using suprahyoid and infrahyoid ultrasonography derived measurements in anesthesiology. Med Ultrason. (2019) 21:83–8. 10.11152/mu-1764 30779836

[12] Wojtczak JA. Submandibular sonography. J Ultrasound Med. (2012) 31:523–8. 10.7863/jum.2012.31.4.523 22441908

[13] Moher D Liberati A Tetzlaff J Altman DG; PRISMA Group. Preferred reporting items for systematic reviews and meta-analyses: the PRISMA statement (Chinese edition). J Chinese Integr Med. (2009) 7:889–96. 10.3736/jcim20090918 PMC3090117 21603045

[14] Liberati A Altman DG Tetzlaff J Mulrow C Gøtzsche PC Ioannidis JP. The PRISMA statement for reporting systematic reviews and meta-analyses of studies that evaluate health care interventions: explanation and elaboration. J Clin Epidemiol. (2009) 62:e1–34. 10.1016/j.jclinepi.2009.06.006 19631507

[15] Moola S Munn Z Tufanaru C Aromataris E Sears K Sfetc R. Chapter 7: Systematic reviews of etiology and risk. In: Aromataris E Munn Z, editors. Joanna Briggs Inst Rev Manual for Evidence Synthesis. The Joanna Briggs Institute, 2020. Available from: https://reviewersmanual.joannabriggs.org/ (accessed at: 10 August 2020).

[16] Review Manager (RevMan) [Computer program]. Version 5.3. Copenhagen: The Nordic Cochrane Centre. The Cochrane Collaboration (2014).

[17] Higgins JPT Thompson SG Deeks JJ Altman DG. Measuring inconsistency in meta-analyses. BMJ. (2003) 327:557–60. 10.1136/bmj.327.7414.557 12958120 PMC192859

[18] Adhikari S Zeger W Schmier C. Pilot study to determine the utility of point-of-care ultrasound in the assessment of difficult laryngoscopy. Acad Emerg Med. (2011) 18:754–8. 10.1111/j.1553-2712.2011.01099.x 21707828

[19] Reddy P Punetha P Chalam K. Ultrasonography - A viable tool for airway assessment. Indian J Anaesth. (2016) 60:807–13. 10.4103/0019-5049.193660 27942053 PMC5125183

[20] Rana S Verma V Bhandari S Sharma S Koundal V Chaudhary SK. Point-of-care ultrasound in the airway assessment: a correlation of ultrasonography-guided parameters to the Cormack–Lehane Classification. Saudi J Anesth. (2018) 12:292–6. 10.4103/sja.SJA_540_17 29628843 PMC5875221

[21] Gupta D Srirajakalidindi A Ittiara B Apple L Toshniwal G Haber H. Ultrasonographic modification of Cormack Lehane classification for pre-anesthetic airway assessment. Middle East J Anesthesiol. (2012) 21:835–42. 23634565

[22] Mohammadi Soltani S Saliminia A Nejatifard N Azma R. Usefulness of ultrasound view of larynx in pre-anesthetic airway assessment: a comparison with cormack-lehane classification during direct laryngoscopy. Anesthesiol Pain Med. (2016) 6:e39566. 10.5812/aapm.39566 28975073 PMC5560580

[23] Petrisor C Szabo R Constantinescu C Prie A Hagau N. Ultrasound-based assessment of hyomental distances in neutral, ramped, and maximum hyperextended positions, and derived ratios, for the prediction of difficult airway in the obese population: a pilot diagnostic accuracy study. Anestezjol Intens Ter. (2018) 50:110–6. 10.5603/AIT.2018.0017 29953573

[24] Wu J Dong J Ding Y Zheng J. Role of anterior neck soft tissue quantifications by ultrasound in predicting difficult laryngoscopy. Med Sci Monit. (2014) 20:2343–50. 10.12659/MSM.891037 25403231 PMC4247231

[25] Ezri T Gewurtz G Sessler DI Medalion B Szmuk P Hagberg C. Prediction of difficult laryngoscopy in obese patients by ultrasound quantification of anterior neck soft tissue. Anesthesia (2003) 58:1101-1105. 10.1046/j.1365-2044.2003.03412.x 14616599 PMC1283106

[26] Komatsu R Sengupta P Wadhwa A Akça O Sessler DI Ezri T. Ultrasound quantification of anterior soft tissue thickness fails to predict difficult laryngoscopy in obese patients. Anaesth Intensive Care. (2007) 35:32–7. 10.1177/0310057X0703500104 17323663

[27] Pinto J Cordeiro L Pereira C Gama R Fernandes HL Assunção J. Predicting difficult laryngoscopy using ultrasound measurement of distance from skin to epiglottis. J Crit Care. (2016) 33:26–31. 10.1016/j.jcrc.2016.01.029 26948251

[28] Yao W Zhou Y Wang B Yu T Shen Z Wu H. Can mandibular condylar mobility sonography measurements predict difficult laryngoscopy? Anesth Analg. (2017) 124:800–6. 10.1213/ANE.0000000000001528 28098589

[29] Chan SMM Wong WY Lam SKT Wong OF Law WSS Shiu WYY. Use of ultrasound to predict difficult intubation in Chinese population by assessing the ratio of the pre-epiglottis space distance and the distance between epiglottis and vocal folds. Hong Kong J Emerg Med. (2018) 25:152–9. 10.1177/1024907917749479

[30] Alessandri F Antenucci G Piervincenzi E Buonopane C Bellucci R Andreoli C. Ultrasound as a new tool in the assessment of airway difficulties. Eur J Anaesthesiol. (2019) 36:1–7. 10.1097/EJA.0000000000000989 31742568

[31] Parameswari A Govind M Vakamudi M. Correlation between preoperative ultrasonographic airway assessment and laryngoscopic view in adult patients: a prospective study. J Anaesthesiol Clin Pharmacol. (2017) 33:353. 10.4103/joacp.JOACP_166_17 29109635 PMC5672513

[32] Falcetta S Cavallo S Gabbanelli V Pelaia P Sorbello M Zdravkovic I. Evaluation of two neck ultrasound measurements as predictors of difficult direct laryngoscopy. Eur J Anaesthesiol. (2018) 35:605–12. 10.1097/EJA.0000000000000832 29889671

[33] Andruszkiewicz P Wojtczak J Sobczyk D Stach O Kowalik I. Effectiveness and validity of sonographic upper airway evaluation to predict difficult laryngoscopy. J Ultrasound Med. (2016) 35:2243–52. 10.7863/ultra.15.11098 27582532

[34] Hui CM Tsui BC. Sublingual ultrasound as an assessment method for predicting difficult intubation: a pilot study. Anesthesia. (2014) 69:314–9. 10.1111/anae.12598 24641637

[35] Yao W Wang B. Can tongue thickness measured by ultrasonography predict difficult tracheal intubation? Br J Anaesth. (2017) 118:601–9. 10.1093/bja/aex051 28403413

[36] Gregor RT. The prepiglottic space revisited: is not significant? Am J Otolaryngol. (1990) 11:161–4. 10.1016/0196-0709(90)90031-P 2382783

[37] Ramsingh D Rinehart J Kain Z Strom S Canales C Alexander B. Impact assessment of perioperative point-of-care ultrasound training on anesthesiology residents. Anesthesiology. (2015) 123:670–82. 10.1097/ALN.0000000000000776 26181338

[38] Yeh L Montealegre-Gallegos M Mahmood F Hess PE Shnider M Mitchell JD. Assessment of perioperative ultrasound workflow understanding: a consensus. J Cardiothorac Vasc Anesth. (2017) 31:197–202. 10.1053/j.jvca.2016.07.008 27686512

[39] Osman A Sum KM. Role of upper airway ultrasound in airway management. J Intensive Care. (2016) 4:52. 10.1186/s40560-016-0174-z 27529028 PMC4983796

[40] Fulkerson JS Moore HM Anderson TS Lowe RF. Ultrasonography in the preoperative difficult airway assessment. J Clin Monit Comput. (2017) 31:513–30. 10.1007/s10877-016-9888-7 27156094

[41] Yentis SM Lee DJH. Evaluation of an improved scoring system for the grading of direct laryngoscopy. Anesthesia. (1998) 53:1041–4. 10.1046/j.1365-2044.1998.00605.x 10023271

[42] Hoefnagel A Yu A Kaminski A. Anesthetic complications in pregnancy. Crit Care Clin. (2016) 32:1–28. 10.1016/j.ccc.2015.08.009 26600441

[43] Mushambi MC Kinsella SM Popat M Swales H Ramaswamy KK Winton AL. Obstetric Anaesthetists' Association and Difficult Airway Society guidelines for the management of difficult and failed tracheal intubation in obstetrics. Anesthesia. (2015) 70:1286–306. 10.1111/anae.13260 26449292 PMC4606761

[44] Das SK Choupoo NS Haldar R Lahkar A. Transtracheal ultrasound for verification of endotracheal tube placement: a systematic review and meta-analysis / Vérification du positionnement du tube endotrachéal par échographie transtrachéale: revue systématique de la littérature et méta-analyse. Can J Anesth Can d'anesthésie. (2015) 62:413–23. 10.1007/s12630-014-0301-z 25537734

[45] Harless J Ramaiah R Bhananker SM. Pediatric airway management. Int J Crit Illn Inj Sci. (2014) 4:65–70. 10.4103/2229-5151.128015 24741500 PMC3982373

[46] Baker PA Feinleib J O'Sullivan EP. Is it time for airway management education to be mandatory? BJA Br J Anaesth. (2016) 117(suppl_1):i13–6. 10.1093/bja/aew129 27276977

