# Supplementary material for: Useful Ultrasonographic Parameters to Predict Difficult Laryngoscopy and Difficult Tracheal Intubation—A Systematic Review and Meta-Analysis
Source: Front Med (Lausanne). 2021 May 28;8:671658. doi: 10.3389/fmed.2021.671658 (PMC8193063; doi:10.3389/fmed.2021.671658)
Supplement: Supplementary file 1 [file Table_1.DOCX]

**Table 1. Synthesis table**

| Article | Study design and methods | Sample selection | Exclusion criteria | Variables and data types | Statistical analysis | US results | Conclusions | Limitations | |
| --- | --- | --- | --- | --- | --- | --- | --- | --- | --- |
| *Ezri, T.*  *2003* | **Objective:** To predict difficult laryngoscopy in morbidly obese patients by US quantification of anterior neck soft tissue.  **Design:** Prospective observational  **Blinding:** Yes.  **Standard characteristics:** age; sex; BMI.  **Clinical evaluation:** history of OSAS; upper teeth pathology; TMD; IIG; neck mobility problems; MMS; neck circumference.  **US measurements:** Skin to anterior commissure of VC (zone 1), skin to anterior aspect of trachea at the level of thyroid isthmus (zone 2) and skin to anterior aspect of trachea at the level of  supraesternal notch (zone 3)  Values obtained as an average of three measurements: at the central axis and 1.5cm to the left and right of the central axis.  **Position:** None specified.  **Laryngoscopy**: with BURP. | **N =** 50  **BMI:** >35 kg/m^2^  **Demographics:** Israeli population  **Inclusion criteria:**  Elective laparoscopic weight reduction surgery under general anaesthesia with intubation.  **Setting:** Israel | Pregnant;  Upper airway abnormalities;  Cervical spine fractures;  Full stomach, hiatus hernia, GERD;  History of difficult laryngoscopy or intubation. | **DV:** CL grades 1 & 2 (easy) versus 3 & 4 (difficult) (nominal/ordinal)  **IV:** Standard characteristics  (numerical/nominal)  **IV:** Clinical screening tests (nominal/ordinal/numerical)  **IV:** Sonographic measures (numerical) | **Data normality test:** Not specified  **Numerical:** Two-sided Student’s *t*-test  **Nominal:** Chi-squared, Fisher’s exact test. | **Difficult laryngoscopy**: n=9  **Significance at** **(p< 0.05):**  - Zone 1: (difficult 2.8±0.27 cm vs. easy 1.75±0.18 cm, p<0.001)  - Zone 3: (difficult 3.3±0.43 cm vs. easy 2.74±0.66 cm, p=0.013) | In morbidly obese patients, US Zone 1 measurement completely separated the patients with difficult and easy laryngoscopies. | | Does not mention the experience of the practitioner performing US evaluation or laryngoscopies.  Same practitioner performing all US evaluations.  Same practitioner performing all clinical evaluations and laryngoscopies. BURP could improve CL grade.  Does not specify patient’s position for US evaluation. |
| *Komatsu, R.*  *2007* | **Objective:** To predict difficult laryngoscopy in morbidly obese patients in the United States by US quantification of anterior neck soft tissue.  **Design:** Prospective observational  **Blinding:** Yes.  **Standard characteristics:** age; sex; race; BMI  **Clinical evaluation:** history of OSAS (diagnosed or suspected); upper teeth pathology; TMD; IIG; TMJ mobility; neck mobility problems; MMS; neck circumference.  **US measurements:** Skin to anterior commissure of VC  Values obtained as an average of three measurements: at the central axis and 1.5cm to the left and right of the central axis.  **Position:** Supine, neutral.  **Laryngoscopy**: BURP allowed for difficult laryngoscopy. CL>3, intubation with bougie  **Intubation**: endotracheal tube preformed with a stylet. | **N =** 64  **BMI:** >35 kg/m^2^  **Demographics** American population  **Inclusion:** Elective surgery under general anaesthesia with intubation.  **Setting:** United States of America | Pregnant;  Removable upper dentures, upper airway abnormalities;  Cervical spine fractures;  Full stomach, significant GERD; History of difficult laryngoscopy | **DV:** CL grades 1 & 2 (easy) versus 3 & 4 (difficult) (nominal / ordinal)  **IV:** Standard characteristics  (numerical/nominal)  **IV:** Clinical screening tests (nominal/ordinal/numerical)  **IV:** Sonographic measures (numerical) | **Data normality test:** Not specified  **Numerical:** Two-sided Student’s *t*-test or Mann-Whitney U test  **Nominal:** Fisher’s exact test or Chi-square  *Multivariate logistic regression* for independent predictors. | **Difficult laryngoscopy:** n=20  **Significance at (p<0.05):** Skin to anterior commissure of VC (difficult 2.04±0.3 cm vs easy 2.23±0.38 cm, p=0.049)  *Multivariate regression analysis* (numerical and nominal): none was an independent predictor of difficult laryngoscopy. | In obese patients in the United States, it does not appear to exist a relationship between the distance from the skin to anterior commissure of VC  and CL grade. | | Same practitioner performing US measurements after evaluating clinical variables.  Small sample heterogeneity.  Does not specify US technique.  No mention as to why the study was stopped by the Data and Safety Monitoring Committee.  Did not include any patients of Middle Eastern descent. |

| Article | | | Study design and methods | | Sample selection | | | | Exclusion criteria | | | Variables and data types | | | Statistical analysis | | | US results | Conclusions | | | | Limitations | | |
| --- | --- | --- | --- | --- | --- | --- | --- | --- | --- | --- | --- | --- | --- | --- | --- | --- | --- | --- | --- | --- | --- | --- | --- | --- | --- |
| *Adhikari, S.*  *2011* | | | **Objective:** To determine the utility of US measurements in distinguishing difficult and easy laryngoscopies.  **Design:** Prospective observational  **Blinding:** Yes.  **Standard characteristics:** age; sex; race; BMI.  **Clinical evaluation:** history of OSAS (diagnosed or suspected); upper teeth pathology; TMD; IIG; TMJ mobility; neck mobility; MMS.  **US measurements:** tongue thickness; skin to hyoid bone, skin to epiglottis at THM level, skin to anterior commissure of VC, skin to anterior aspect of trachea at the level of thyroid isthmus and at supraesternal notch level.  Values obtained as an average of three measurements: at the central axis and 1cm to the left and right of the central axis.  **Position:** Supine, neutral. | | **N =** 51  **Demographics:** whites and african americans.  **Inclusion:** Adults (≥19); Elective surgery requiring intubation.  **Setting:** United States of America academic hospital. | | | | Facial fractures; Maxillofacial abnormalities; Tumours; Cervical spine fractures; Tracheostomy tube;  Unable to  consent;  Regional anaesthesia;  Use of laryngeal mask. | | | **DV:** CL grades 1 & 2 (easy) versus 3 & 4 (difficult) (nominal / ordinal)  **IV:** Standard characteristics  (numerical/ nominal)  **IV:** Clinical screening tests (nominal/ordinal/numerical)  **IV:** Sonographic measures (numerical) | | | **Data normality:**  Shapiro-Wilk test  **Numerical:** Two-sided Student’s *t*-test  **Nominal:** Fisher’s exact test  Did not adjust for multiple comparisons.  *Spearman’s rank correlation* | | | **Difficult laryngoscopy:** n=6  **Significance at** **(p<0.05):**  - Skin to hyoid bone (difficult 1.69cm (95% CI: 1.19 to 2.19) vs easy 1.37cm (1.27 to 1.46)).  - Skin to epiglottis at THM level (difficult 3.47cm (2.88 to 4.07) vs easy 2.37cm (2.29 to 2.44)). | A 2.8 cm US measurement at thyrohyoid membrane level completely separated the  patients with difficult and easy laryngoscopies. | | | | Same practitioner performing all US evaluations.  Not all results reported.  No p values.  Small sample size (cutoff not validated).  Investigators were not blinded to the study hypothesis.  Different anesthesia providers.  No standardized laryngoscopy procedure. | | |
| *Gupta D.*  *2012* | | | **Objective:** To compare and correlate the US view of the airway and the CL classification.  **Design:** Prospective, cross-sectional.  **Blinding:** Yes.  **Clinical evaluation:** Mallampati classification  **US measurements:** Pre-E, mVC, Pre-E/mVC.  **Position:** supine, extended. | | **N=** 49  **Demographics:** not specified.  **Inclusion:** Adults (18-80), scheduled to undergo general anaesthesia with direct laryngoscopy and tracheal intubation.  **Setting:** United States. | | | | Head and Neck anatomic pathology; Limited mouth opening (too small to pass Macintosh blade 4); Edentulous; Inability to extend neck >30º. | | | **DV:** CL grades 1 & 2 (easy) versus 3 & 4 (difficult) (nominal/ordinal)  **IV:** Clinical screening tests (ordinal)  **IV:** Sonographic measures (numerical) | | | **Bland-Altman** with limits of agreement for Mallampati and CL.  *Regression coefficients*  *ROC curve* | | | **Difficult laryngoscopy:** n=12  **Regression coefficient (p<0.05):**  - E-VC and CL: 0.966  - Pre-E and CL: 0.595  - Pre-E/E-VC: 0.495 | | The extremes of the CL grade (grade 1 and 3) will be better assessed and predicted by Pre-E/E-VC than the CL  grade 2. | | | Small sample size.  No CL 4 patients.  Same practitioner performing all US evaluations.  23 subjects excluded due to lack of operator proficiency. | | |
| *Wojtczak, J. A.*  *2012* | | | **Objective:** To evaluate the feasibility of submandibular US to predict difficult laryngoscopy in obese patients with a large neck circumference.  **Design:** Retrospective observational  **Blinding:** Not mentioned.  **Standard characteristics:** age; sex; BMI  **Clinical evaluation:** MMS; neck circumference.  **US measurements:** HMD (neutral and hyperextended position); HDMR2; tongue volume; floor of the mouth muscles volume.  **Position:** Supine, neutral and extended. | | **N =** 12  **BMI:** >30 kg/m^2^  **Demographics:** American population  **Inclusion:** Adults undergoing preoperative anaesthesia evaluation;  history of easy or difficult laryngoscopy**.**  **Setting:** United States of America | | | | Not specified. | | | **DV:** CL grades 1 & 2 (easy) versus 3 & 4 (difficult) (nominal / ordinal) (anaesthesia records)  **IV:** Standard characteristics  (numerical)  **IV:** Clinical screening tests (ordinal/numerical)  **IV:** Sonographic measures (numerical) | | | **Data normality test:** Not specified  **Numerical:** Two-sided Student’s *t*-test (unpaired) | | | **Difficult laryngoscopy**: n=6  **Significance at** **(p< 0.05):**  - HMDR2 (difficult 1.02±0.01 vs. easy 1.14±0.02, p<0.002)  - Mean HMD in hyperextended position (difficult 5.26±0.58 cm vs. easy 6.55±0.41 cm, p<0.01) | | Preoperative assessment of the HMDR2 may predict difficult laryngoscopy resulting in difficult intubation in obese patients. | | | Neck circumference was not evaluated to select the sample.  Blinding not mentioned.  Exclusion criteria not mentioned.  Experience of the practitioner performing US evaluation or laryngoscopies not mentioned.  Some p values not mentioned.  Small sample size. | | |
| Article | Study design and methods | | | | | Sample selection | | Exclusion criteria | | | Variables and data types | | | Statistical analysis | | | US results | | | | | Conclusions | | | Limitations |
| *Hui, C. M.*  *2014* | **Objective:** To determine the feasibility and accuracy of sublingual US to predict a difficult airway.  **Design:** Prospective observational.  **Blinding:** Yes.  **Standard characteristics:** age; sex; BMI; ASA physical status; type of surgery procedure.  **Clinical evaluation:** TMD; IIG; neck extension; size of mandible; MMS; dentition.  **US measurements:** Visibility of hyoid bone through the floor of the mouth.  **Position:** Sitting, neutral. | | | | | **N =** 100  **Demographics:** Canadian population  **Inclusion:** Adults (>17) recruited on the day of elective surgery, requiring intubation.  **Setting:** Canada | | Intubation without direct laryngoscopy; Upper airway abnormalities;  Lack of neuromuscular blockade. | | | **DV:** CL grades 1 & 2 (easy) versus 3 & 4 (difficult) (nominal/ordinal)  **IV:** Clinical screening tests (nominal/ordinal/numerical)  **IV:** Sonographic measures (nominal) | | | **Nominal:** Fisher’s exact test.  *Diagnostic validity profile* | | | **Difficult laryngoscopy**: n=11  **Significance at:** Hyoid bone not visible (p<0.0001).  **Diagnostic validity profile:**  *Sensitivity:* 73%  *Specificity:* 97%  *Positive Likelihood Ratio:* 21.6 *Negative Likelihood Ratio:* 0.28 | | | | | Hyoid bone not visible on sublingual US can be a predictor of a difficult laryngoscopy. | | | Same practitioner performing all US evaluations.  Patients placed US probe.  Different practitioners performed the clinical evaluation and laryngoscopy |
| *Wu, J.*  *2014* | **Objective:** To determine if US measurements of anterior neck soft tissue can be used to predict difficult laryngoscopy.  **Design:** Prospective observational.  **Blinding:** No.  **Standard characteristics:** Age; sex; weight; height; BMI  **Clinical evaluation:** TMD; IIG; MMS.  **US measurements:** Skin to hyoid bone, skin to epiglottis at THM level and skin to anterior commissure of VC.  **Laryngoscopy**: No BURP  **Position:** Supine, neutral. | | | | | **N =** 203  **Demographics:** Chinese Han population  **Inclusion:** Adults (20-65), scheduled to undergo general anaesthesia.  **Setting:** China | | History of facial, cervical, pharyngeal and epiglottis surgery or trauma;  Most teeth lost;  Arthritis. | | | **DV:** CL grades 1 & 2 (easy) versus 3 & 4 (difficult) (nominal/ ordinal)  **IV:** Standard characteristics  (numerical/nominal)  **IV:** Clinical screening tests (ordinal/numerical)  **IV:** Sonographic measures (numerical) | | | **Data normality test:** Not specified  **Numerical:** Two-sided Student’s *t*-test.  **Nominal:** Fisher’s exact test or Chi-square test.  *Pearson correlation* (least-squares regression)  *ROC curve* (DeLong method; Youden index). | | | **Difficult laryngoscopy:** n=28  **Significance at** **(p<0.05):**  - Skin to hyoid bone (difficult 1.51±0.27cm vs easy 0.98±0.26cm, p<0.0001).  - Skin to epiglottis at THM level (difficult 2.39±0.34cm vs easy 1.49±0.39cm, p<0.0001).  - Skin to anterior commissure of VC (difficult 1.30±0.31cm vs easy 0.92±0.20cm, p<0.0001).  **Strong positive correlation:** Skin to hyoid bone and thyrohyoid membrane (r= 0.74)  **Optimal cutoff values / AUC:** (sensitivity and specificity)  - Skin to hyoid bone: 1.28cm (85.7%, 85.1%) / 0.92  - Skin to epiglottis at THM level: 1.78cm (100%, 66.3%) / 0.90  - Skin to anterior commissure of VC: 1.1cm (75%, 80.6%) / 0.85 | | | | | Skin to hyoid bone measured by US (>1.28cm), to epiglottis at THM level (>1.78cm), and to anterior commissure of VC (>1.1cm) are independent predictors of difficult laryngoscopy. | | | Same practitioner performing all US evaluations.  No blinding. |
| *Pinto, J.*  *2016* | | **Objective:** To evaluate the use of US-measured distance from skin to epiglottis (DSE) for difficult laryngoscopy prediction.  **Design:** Prospective observational  **Blinding:** Yes.  **Standard characteristics:** age; sex; weight; height.  **Clinical evaluation:** MMS; IIG; TMD; neck circumference; Naguib score.  **US measurements:** skin to epiglottis at THM level (DSE); MMS-DSE decision tree  Values obtained as an average of three measurements: at the central axis and the left and right extremities of epiglottis.  **Position:** Supine, neutral. | | | | **N=** 74  **Demographics:** Portuguese population  **Inclusion:** Adults requiring intubation for elective surgery.  **Setting:** Hospital Center Tondela-Viseu, Portugal. | | Facial/cervical fractures; Maxillofacial abnormalities; Cervical tumors;  Goiter; Tracheostomy tube;  Morbid obesity;  Pregnant;  Unable to consent; | | | **DV:** CL grades 1 & 2 (easy) versus 3 & 4 (difficult) (nominal / ordinal)  **IV:** Standard characteristics  (numerical/ nominal)  **IV:** Clinical screening tests (ordinalnumerical)  **IV:** Sonographic measures (numerical) | | | **Data normality test:** Not specified  **Numerical:** Two-sided Student’s *t*-test.  **Nominal:** Fisher’s exact test or Chi-square test.  *Diagnostic validity profile*  **Cutoff for DSE:** Youden’s index. | | | **Difficult laryngoscopy:** n= 17  **Significance at** **(p<0.05):**  - Skin to epiglottis at THM level (difficult 2.825±0.443cm vs easy 2.332±0.386cm, p<0.001).  **Performance evaluation**  The skin to epiglottis at THM level has the highest average accuracy, specificity, and PPV. | | | | | Laryngoscopy will be predicted as difficult for patients with a distance from skin to epiglottis at THM level ≥2.75cm and easy otherwise. | | | Same practitioner performing all US evaluations.  Does not mention the experience of the sonographer. |
| Article | | Study design and methods | | Sample selection | | | Exclusion criteria | | | Variables and data types | | | Statistical analysis | | | US results | | | | | Conclusions | | | Limitations | |
| *Andruszkiewicz P.*  *2016* | | **Objective**: To evaluate the effectiveness of 9 airway US parameters imaged from the submandibular view as predictors of difficult laryngoscopy.  **Design**: Prospective observational.  **Blinding**: Yes.  **Standard characteristics**: age; sex; BMI.  **Clinical Evaluations:** MMS; TMD; IIG; ULBT.  **US measurements:** HMD neutral, HMD extended, HMDR2, tongue cross-sectional area, tongue width, tongue volume, tongue thickness-to-oral cavity height ratio, floor of the mouth muscle cross-sectional area, floor of the mouth muscle volume  **Position**: Supine**,** neutral and extended. | | **N =** 199  **Demographics:** Polish population  **Inclusion criteria:**  Adults (>18); Elective surgery under general anaesthesia with intubation.  **Setting**: University Teaching Hospital, Poland | | | Difficult intubation predicted by medical history; Gross anatomic abnormalities; Sonographer unavailable. | | | **DV:** CL grades 1 & 2 (easy) versus 3 & 4 (difficult) (nominal / ordinal)  **IV:** Standard characteristics  (numerical/ nominal)  **IV:** Clinical screening tests (ordinal/numerical)  **IV:** Sonographic measures (numerical) | | | **Data normality test:** skewness.  **Numerical:** Two-sided Student’s *t*-test (unpaired).  **Nominal:** Chi-squared, Fisher’s exact test  *Multivariate Logistic Regression*  *ROC curve* (DeLong) | | | **Difficult laryngoscopy**: n=22.  **Significance at (p< 0.05):**  - HMD in neutral (difficult 3.99±0.56cm vs easy 4.32±0.42cm, p=0.0014, AUC= 0.660)  - HMD in extension (difficult 4.28±0.64cm vs easy 4.82±0.46cm, p=0.0009, AUC= 0.758)  - HMDR2 (difficult 1.07±0.08 vs easy 1.12±0.07, p=0.0022, AUC= 0.710)  - Tongue cross-sectional area (difficult 23.1±3.57cm^2^ vs easy 21.6±3.09 cm^2^, p=0.333, AUC= 0.622)  - Tongue volume (difficult 121.7±27.1cm^3^ vs easy 111.2±22.1 cm^3^, p=0.415, AUC= 0.626)  **Multivariate Logistic Regression (AUC= 0.898):** HMD in extension (p<0.0001), tongue volume (0.0003) | | | | | HMD in extension and tongue volume had the highest impact on the diagnostic validity profile.  HMD in extension had the highest AUC value followed by tongue volume. The combination  of tests improved the diagnostic validity profile. | | | Same practitioner performing all US measurements.  Easy and difficult laryngoscopy groups were uneven.  Tongue volume calculation formula still not established.  Exclusion of patients with a predicted difficult intubation.  Did not have any patient with a known pathologic tongue enlargement. | |
| *Mohammadi, S.*  *2016* | | **Objective:** To compare and correlate the US view of the larynx with CL classification during direct laryngoscopy under general anaesthesia.  **Design:** Cross-sectional observational.  **Blinding:** Not mentioned.  **Standard characteristics**: Age; sex; weight; height; BMI; ASA.  **Clinical evaluation:** Mallampati classification.  **US measurements:** Pre-E; E-VC; Pre-E/E-VC ratio.  **Position:** Supine, extended. | | **N =** 47  **Demographics:** Iranian population  **Inclusion:** Adults (18-70); Elective surgery under general anaesthesia with direct laryngoscopy and intubation.  **Setting:** Tehran, Iran | | | Edentulous; Cranial-cervical region abnormalities;  Fractures of maxillofacial or cervical bones;  IIG <4 cm; inability to hyperextend the neck;  BMI ≥40 kg/m^2^. | | | **DV:** CL grades (ordinal)  **IV:** Clinical screening test (ordinal)  **IV:** Sonographic measures (numerical) | | | **Data normality test:** Not specified  **Numerical:** Two-sided Student’s *t*-test.  **Ordinal:** Chi-squared test.  *ROC curve.*  *Diagnostic validity profile.*  *Correlation coefficient and regression analyses.* | | | **CL3**: n=6; **CL4:** n=0  No significant results (p>0.05)  Weak correlation between US measured pre-E/E-VC ratio and CL grading (87.5% sensitivity and 30% specificity). | | | | | US measurement criteria are not accurate for airway evaluation before anaesthesia. | | | Does not mention the experience of the practitioner performing laryngoscopy.  US performed by a resident.  Small sample heterogeneity (no CL 4 patients)  Not all results reported. | |
| *Reddy, P.*  *2016* | | **Objective**: To determine the utility of US in predicting CL grade.  **Design:** Prospective observational.  **Blinding:** Yes.  **Standard characteristics:** BMI.  **Clinical evaluation:** MMS; TMD; SMD  **US Measurement:** Skin to hyoid bone and skin to anterior commissure of VC; Pre-E/E-VC.  **Position:** Supine, extended. | | **N** = 100  **Demographics**: Asian population  **Inclusion**: Adults (18-70); BMI 14.2-39.0 kg/m^2^; Elective surgery under general anaesthesia with direct laryngoscopy and intubation.  **Setting:** Tertiary care centre, India. | | | Requiring rapid sequence intubation; Cervical spine and airway pathology; Scheduled for fibreoptic tracheal intubation; Uncooperative; Pregnant. | | | **DV:** CL grades 1 & 2 (easy) versus 3 & 4 (difficult) (nominal / ordinal)  **IV:** Clinical screening test (nominal/ordinal)  **IV:** Sonographic measures (numerical) | | | **Nominal:** Chi-squared test.  *ROC curve.*  *Diagnostic validity profile.*  *Correlation and regression coefficient.* | | | **Difficult laryngoscopy:** n = 14  **Significance at (p<0.05):**  - Skin to anterior commissure of VC (p=0.014; CL 3 = 0.35±0.18cm)  - Pre-E/E-VC (p=0.044; CL 3 = 1.29±0.44)  **Optimal cutoff values / AUC:** (sensitivity and specificity)  - Skin to anterior commissure of VC: 0.23 cm (85.7%; 57%) / 0.73 | | | | | Skin to anterior commissure of VC >0.23 cm is a potential predictor of difficult intubation (highest sensitivity).  Pre-E/E-VC ratio was useful in predicting a difficult intubation but has a low to moderate predictive value. | | | Same practitioner performing all preoperative assessments.  No CL 4 patients.  Abnormal dentition was not excluded.  Only 6% were obese. | |

| Article | Study design and methods | Sample selection | Exclusion criteria | Variables and data types | Statistical analysis | US results | Conclusions | Limitations |
| --- | --- | --- | --- | --- | --- | --- | --- | --- |
| *Parameswari, A.*  *2017* | **Objective:** To find correlation between preoperative US airway assessment parameters and CL grade.  **Design:** Prospective observational.  **Blinding:** Yes.  **Standard characteristics:** Age; sex; BMI.  **Clinical evaluation:** TMD; IIG; MMS; neck circumference.  **US measurements:** anteroposterior thickness of the geniohyoid muscle, skin to hyoid bone and skin to epiglottis at THM level, HMD, cross‑sectional area and volume of the muscles of the floor of the mouth, volume of the tongue.  **Position:** Supine, neutral. | **N =** 130  **Demographics:** Indian population  **Inclusion criteria:**  Adults (18-60); elective surgery under general anaesthesia with intubation.  **Setting:** India | Any feature of difficult airway such as maxillofacial anomalies;  Restricted neck movements;  BMI >40 kg/m^2^; Limited mouth opening. | **DV:** CL grades 1 & 2 (easy) versus 3 & 4 (difficult) (nominal / ordinal)  **IV:** Clinical screening tests (ordinal/numerical)  **IV:** Sonographic measures (numerical) | **Nominal:** Chi-squared, Fisher’s exact test.  *Diagnostic validity profile*  *ROC curve* | **Difficult laryngoscopy**: n=12  **Significance at (p< 0.05):**  - Skin to epiglottis at THM level: 75% sensitivity, 63.6% specificity. (AUC= 0.693)  - Volume of the tongue: 66.7% sensitivity, 62.7% specificity.  - Skin to hyoid bone: 58.3% sensitivity, 56.8% specificity.  - Volume of the floor of the mouth: 50% sensitivity, 55.9% specificity. | Skin to epiglottis at THM e is the most sensitive and specific.  US parameters have better NPV than PPV. | Cutoff points  for US variables not established.  Does not mention the experience of the practitioner performing US evaluation or laryngoscopies.  No p values.  Not all results reported.  Unclear statistical analysis. |
| *Yao, W.*  *(27)* | **Objective:** To evaluate the predictive value of US measured tongue thickness for predicting difficult laryngoscopy and intubation.  **Design:** Prospective observational  **Blinding:** Yes.  **Standard characteristics:** Age; sex; BMI.  **Clinical evaluation:** MMS; IIG; TMD; MMS to TMD ratio.  **US measurements:** Tongue thickness; and tongue thickness to TMD ratio.  **Position:** Supine, extended.  **Laryngoscopy**: BURP allowed. | **N =** 2254  **Demographics:** Chinese Han population  **Inclusion:** Adults (18 - 90); elective surgery with general anaesthesia requiring intubation.  **Setting:** Hospital of Wannan Medical College, China | Upper airway anatomical deformity trauma or tumor;  Difficult  airway history that required the patient to be awake during tracheal intubation; Subglottic airway stenosis;  Dropouts; Modified anaesthesia protocol or cancellation of intubation for a nondifficult-airway reason; Missing patient’s data. | **DV:** Tracheal intubation difficulty (difficult: > 2 attempts, >10 min, or required  an alternative technique) (nominal)  **DV:** Laryngoscopy difficulty: CL grades 1 & 2 (easy) versus 3 & 4 (difficult) (nominal / ordinal)  **IV:** Standard characteristics  (nominal/numerical)  **IV:** Clinical screening tests (ordinal/numerical)  **IV:** Sonographic measure (numerical) | **Data normality test:** Not specified  **Numerical:** Two-sided Student’s *t*-test or Mann-Whitney U test.  **Nominal:** Fisher’s exact test or Chi-square test.  *Multivariable logistic regression.*  *ROC curve* (Youden’s index).  *Diagnostic validity profile* of independent predictors.  *Pearson/ Spearman correlation.*  Evaluation of reliability between the 2 sonographers. | **Difficult laryngoscopy**: n = 142  **Difficult intubation:** n= 51.  **Significance at** **(p<0.05):**  - Tongue thickness (difficult *laryngoscopy* 6.2±0.5cm vs easy 5.8±0.5cm, p<0.001, AUC=0.69; difficult *intubation* 6.4±0.4cm vs easy 5.9±0.5cm, p<0.001, AUC=0.78).  - Tongue thickness/TMD (difficult *laryngoscopy* 0.9±0.13 vs easy 0.79±0.11, p<0.001, AUC=0.75; difficult *intubation* 0.94±0.10 vs easy 0.80±0.11, p<0.001, AUC=0.86).  **Logistic regression** (AUC=0.87 for difficult laryngoscopy/ 0.95 for difficult intubation): tongue thickness >6.1/6.0cm.  **Optimal cutoff value:**  (sensitivity; specificity)  Tongue thickness:  - difficult laryngoscopy: 6.0cm (63%; 66%)  - difficult intubation: 6.1cm (75%; 72%).  Ratio of tongue thickness to TMD: 0.87  - difficult laryngoscopy (63%; 81%)  - difficult intubation (84%; 79%) | Tongue thickness >6.0 cm is an independent predictor for difficult laryngoscopy.  Tongue thickness measured by US and its ratio to TMD present significant capacities to predict difficult laryngoscopy and difficult tracheal intubation. |  |

| Article | Study design and methods | Sample selection | | | Exclusion criteria | | Variables and data types | | Statistical analysis | | US results | Conclusions | | Limitations | |
| --- | --- | --- | --- | --- | --- | --- | --- | --- | --- | --- | --- | --- | --- | --- | --- |
| *Yao W.*  *(24)* | **Objective:** To observe the correlation between US measured mandibular condylar mobility, and other related assessment results, and their capacity for predicting difficult laryngoscopy.  **Design:** Prospective, observational.  **Blinding:** Yes.  **Standard characteristics:** Sex; age; BMI.  **Clinical evaluation:** IIG; ULBT; mandibular protrusion distance; condyle-tragus distance <1 finger; TMD; MMS.  **US measurements:** condylar translation**.**  **Position:** sitting. | **N** = 484  **Demographics:** Chinese population  **Inclusion:** Adults (18-90); Elective surgery with general anaesthesia requiring intubation.  **Setting**: China. | | | Maxillofacial trauma or malformation; Maxillofacial, oral or tongue cancer; Neck tumor compression;  Difficult  airway history that required the patient to be awake during tracheal intubation. | | **DV:** CL grades 1 & 2 (easy) versus 3 & 4 (difficult) (nominal / ordinal)  **IV:** Standard characteristics  (nominal/numerical)  **IV:** Clinical screening tests (nominal/numerical)  **IV:**  Sonographic measure (numerical) | | **Data normality test:** Not specified  **Numerical:** Mann-Whitney U test.  **Nominal:** Chi-squared, Fisher’s exact test  *Spearman correlation*  *ROC Curve* (Youden Index)  *Diagnostic validity profile*  *Multivariate Logistic Regression*  Evaluation of reliability between the 2 sonographers. | | **Difficult laryngoscopy**: n = 41  **Difficult intubation**: n = 5  **Significance at (p<0.05):**  - Condylar translation (difficult *laryngoscopy* 0.92±0.17cm vs easy 1.37±0.25cm; p<0.001)  **Optimal cutoff value / AUC:** (sensitivity and specificity)  - Condylar translation: 1 cm (81% and 91%) / 0.93  Condylar translation was correlated with CL grade.  **Logistic regression** (AUC= 0.97): Condylar translation ≤1cm proved to be an independent predictor. | Condylar translation (≤1 cm)  is a meaningful TMJ mobility evaluation for predicting difficult  laryngoscopy (highest AUC). | | Difficulty in enrolling all who met the inclusion criteria.  Difficulty in randomly selecting the subjects.  Difficulty in identifying clinical TMJ disorders.  Patients with a baseline decrease TMJ function were not excluded.  Condylar translation evaluation method not standardized. | |
| *Chan, S.*  *2018* | **Objective:** To investigate the accuracy of the Pre-E/E-VC ratio measured by US to predict potential difficult airway.  **Design:** Prospective observational.  **Blinding:** Yes.  **Standard characteristics:** Age; sex; weight; height; BMI.  **Clinical evaluation:** TMD; MMS; neck circumference.  **US measurements:** Pre-E; distance from epiglottis to anterior VC (aVC), to the midpoint between the anterior and posterior vocal cords (mVC), and to the posterior vocal cords (pVC); Pre-E/aVC, Pre-E/mVC, Pre-E/pVC ratios.  **Position:** Supine, extended. | **N =** 113  **Demographics:** Chinese population.  **Inclusion:** Adults (≥18); Elective surgery requiring general anaesthesia with direct laryngoscopy and intubation.  **Setting:** Tuen Mun Hospital, China. | | | Limited mouth opening;  Head and neck pathologies (eg. Tumors and facial fracture or cervical spine instability); Tracheostomy;  Limited neck extension (<30°); Refused to participate  in the study. | | **DV:** CL grades 1 & 2a (easy) versus 2b, 3 & 4 (difficult) (nominal / ordinal)  **IV:** Standard characteristics  (nominal/numerical)  **IV:** Clinical screening tests (ordinal/numerical)  **IV:** Sonographic measures  (numerical)  Evaluation of reliability between the 2 sonographers. | | *ROC curve* (minimum distance to upper left corner)  *Diagnostic validity profile* | | **Difficult laryngoscopy**: n = 39  **Optimal cutoff value / AUC:** (sensitivity and specificity)  - Pre-E/aVC ratio: 1 (79.5%; 39.2%) / 0.648. | Pre-E/aVC ratio (>1) had better accuracy than pre-E/mVC and pre-E/ pVC. | | The inter and intra-rater reliabilities of the airway assessment by the anaesthetists were not assessed.  5 patients excluded because video laryngoscopes were used (difficult airway suspected?) | |
| *Falcetta, S.*  *2018* | **Objective:** To predict clinically unexpected difficult airway by US measurement of skin to epiglottis at THM level.  **Design:** Prospective observational.  **Blinding:** Yes.  **Standard characteristics:** Age; sex; BMI.  **US measurements:** skin to anterior commissure of VC and skin to epiglottis at THM level; pre-epiglottic area (PEA).  Values obtained as an average of three measurements: at the central axis and 1cm to the left and right.  **Laryngoscopy**: BURP allowed  **Position:** Supine, neutral. | **N =** 301  **Demographics:** Italian population  **Inclusion:** Adults (≥18 years); elective surgery under general anaesthesia with intubation.  **Setting:** Teaching hospital in Italy | | | Clinically predicted difficult intubation (i.e. IIG <3cm, TMD <6cm); History of previous difficult intubation; Limited cervical spine motility, maxillofacial anomalies;  Rapid sequence induction of anaesthesia; Uncooperative; Pregnant. | | **DV:** CL grades 1 & 2a (easy) versus 2b to 4 (difficult) (nominal / ordinal)  **IV:** Sonographic measures (numerical) | | **Data normality test:** Kolmogorov-Smirnov’s test  **Numerical:** Two-sided Student’s *t*-test  *ROC curve*. | | **Difficult laryngoscopy**: n=28  **Optimal cut-off value / AUC:**  (sensitivity and specificity)  - Skin to epiglottis at THM level: 2.54 cm (82%; 91%) / 0.906  - PEA: 5.04cm^2^ (85%; 88%) / 0.930 | US measured the distance from skin to epiglottis at THM level (>2.54cm) and PEA (>5.04cm^2^) might be predictors of difficult direct laryngoscopy. | | Exclusion of patients with a predicted difficult intubation.  Not all results reported. | |
| Article | Study design and methods | | Sample selection | Exclusion criteria | | Variables and data types | | Statistical analysis | | US results | | | Conclusions | | Limitations |
| *Rana, S.*  *2018* | **Objective:** To determine the efficacy of US measurements in predicting difficult airway.  **Design:** Prospective observational.  **Blinding:** Yes.  **Standard characteristics:** age; sex; BMI; ASA.  **Clinical evaluation:** IIG; MMS; TMD; neck mobility.  **US measurements:** E-VC; Pre-E; Pre-E/E-VC; HMD (neutral and extended); HMDR2.  **Position:** Supine, neutral and extended. | | **N =** 120  **Age:** 20-60 years  **BMI:** <25 kg/m^2^  **Demographics:** Indian population  **Inclusion:** Elective surgery under general anaesthesia with intubation.  **Setting:** India. | IIG <3 cm; Edentulous, head and neck anatomical pathologies; Altered level of consciousness,  inability to follow commands. | | **DV:** CL grades 1 & 2 (easy) versus 3 & 4 (difficult) (nominal/ ordinal)  **IV:** Standard characteristics  (numerical/ordinal/ nominal)  **IV:** Sonographic measures (numerical). | | **Nominal:** Chi-squared test.  *ROC curve* (Youden’s index).  *Diagnostic validity profile.* | | **Difficult laryngoscopy**: n=28  **Significance at** **(p< 0.05):**  - Pre-E/E-VC ratio: CL1 1.33±0.335; CL2 1.62±0.264; CL3 1.87±0.243; CL4 2.22±0.29 (p<0.001)  - HMDR2: CL1 1.11±0.35; CL2 1.12±0,29; CL3 1.07±0.39; CL4 1.04±0.01 (p<0.001)  **Optimal cutoff values / AUC:** (sensitivity, specificity)  - Pre-E/E-VC ratio: 1.77 (82%, 80%) / 0.868  - HDMR2: 1.085 (75%,85.3%) / 0.871  **Strong positive correlation:** Pre-E/E-VC ratio to CL.  **Strong negative correlation:** HDMR2 ratio to CL. | | | Pre-E/E-VC has high predictability with cutoff value >1.77 for predicting difficult laryngoscopy. HMDR is a potential predictor of difficult laryngoscopy, being a sensitive indicator if ≤1.085. | | Restricted inclusion of patients with BMI <25 kg/m^2^  Unclear statistical analysis.  Experience of the practitioner performing US evaluation not mentioned. |
| *Petrișor C.*  *2018* | **Objective**: To evaluate the performance of the US measured HMD, HMDR1 and HMDR2 in the prediction of CL grades 3 or 4 during direct laryngoscopy.  **Design:** Prospective observational.  **Blinding:** Not mentioned.  **Standard Characteristics:** Sex; age; BMI.  **Clinical evaluation:** MMS; ULBT.  **US measurements:** HMD in the neutral, ramped, extended positions; skin to epiglottis at THM level.  **Position:** Supine; neutral, ramped and extended. | | **N =** 25  **BMI**: > 40Kg/m^2^  **Demographics**: Romanians.  **Inclusion:** Elective surgery under general anaesthesia with intubation.  **Setting:** Romania. | Planned videolaryngoscope;  Rapid sequence induction with the external laryngeal manipulation;  Refused to participate in the study. | | **DV:** CL grades 1 & 2 (easy) versus 3 & 4 (difficult) (nominal / ordinal)  **IV:** Standard characteristics  (nominal/numerical)  **IV:** Clinical screening tests (ordinal)  **IV:** Sonographic measures (numerical). | | *ROC curve*  *Diagnostic validity profile* | | **Difficult laryngoscopy**: n = 4  **Significance at** **(p< 0.05):**  - HMD ramped (difficult 4.53±0.1cm vs. easy 5.17±0.28cm; p=0.03)  - HMD extension (difficult 4.9±0.22cm vs. easy 5.8±0.42cm; p=0.01)  - HMDR1 (difficult 1.12±0.001 vs. easy 1.2±0.01; p=0.02)  - HMDR2 (difficult 1.21±0.0005 vs. easy 1.34±0.01; p=0.0002).  **Optimal cutoff values / AUC:** (sensitivity and specificity)  - HMD ramped: 4.97cm (100%; 61.9%) / 0.82  - HMD extended 5.5cm (100%; 71.4%)/ 0.87  - HMDR1: 1.12 (75%; 76.2%) / 0.75  - HMDR2: 1.23 (100%; 90.5%) / 0.92 | | | The inability to expand HMD >23%, allowed to identify all patients with difficult laryngoscopy.  HMDR2 has better accuracy in predicting difficult laryngoscopy in the obese population  compared to the other US measurements. | | Small sample size  Unclear statistical analysis. |
| *Alessandri F.*  *2019* | **Objective:** To evaluate the ability of preoperative US assessment of neck anatomy in predicting DMV and difficult laryngoscopy.  **Design:** Prospective observational.  **Blinding:** Yes.  **Standard characteristics:** Age; sex; height; weight; BMI; ASA.  **Clinical evaluation:** Mallampati score; TMD; IIG; ULBT; dentition; history of OSAS requiring CPAP; surgery.  **US measurements:** Skin to hyoid bone, skin to epiglottis at THM level, skin to anterior commissure of VC, skin to anterior aspect of trachea at the level of thyroid isthmus and skin to anterior aspect of trachea at the level of supraesternal notch.  **Position:** Supine, neutral. | | **N=** 194  **Demographics:** Italian population  **Inclusion:** Adults (>18), requiring intubation for elective ear, nose and throat surgery.  **Setting:** Teaching hospital, Italy. | Facial, cervical, pharyngeal and epiglottic cancer or trauma;  Neck masses; Previous thyroid surgery; Tracheostomy; Pregnancy | | **DV:** CL grades 1 & 2a (easy) versus 2b to 4 (difficult) (nominal / ordinal)  **IV:** Standard characteristics  (numerical/ nominal)  **IV:** Clinical screening tests (nominal/ordinal)  **IV:** Sonographic measures (numerical) | | *Pearson correlation*  *ROC Curve* | | **Difficult laryngoscopy:** n = 34.  **-** Skin to hyoid bone (difficult 1.08±0.41cm vs. easy 0.86±0.28cm)  - skin to epiglottis at THM level (difficult 0.91±0.28cm vs. easy 0.78±0.22cm)  **Pearson correlation \| AUC:**  - Skin to hyoid bone: 0.345 \| 0.660  - Skin to anterior aspect of trachea at the level of thyroid isthmus: 0.301 \| 0.584  - Skin to epiglottis at THM level: 0.273 \| 0.644  - Skin to anterior aspect of trachea at the level of supraesternal notch: 0.210 \| 0.609  - Skin to anterior commissure of VC: 0.144 \| 0.586 | | | There is a relationship between the US measurements assessed and difficult laryngoscopy. | | Same practitioner performing all US measurements.  Skin to hyoid bone cutoff value not defined. |

**Abbreviations**: US, ultrasound; ASA, American Society of Anaesthesiologists; BMI, body mass index; BURP, backward, upward and rightward pressure; TMD, thyromental distance; THM, thyrohyoid membrane; IIG, inter-incisor gap; MMS, modified Mallampati score; ULBT, upper lip bite test; GERD, gastroesophageal reflux; OSAS, obstructive sleep apnoea syndrome; CL, Cormack-Lehane classification; TMJ, temporo-mandibular joint; HMD, hyomental distance; HMDR1, HMD in the ramped position to that in the neutral position ratio; HMDR2, HMD in the extended position to that in the neutral position ratio; Pre-E, pre-epiglottic distance; E-VC, distance from epiglottis to the midpoint of the distance between vocal cords; aVC, distance from the epiglottis to the anterior vocal cords; mVC, distance from the epiglottis to the midpoint between the anterior and posterior vocal cords; pVC, distance from the epiglottis to the posterior vocal cords; SMD, distance from suprasternal notch to mentum with the neck fully extended; DMV, difficult mask ventilation; DV, dependent variable; IV, independent variable; ROC, receiver operating characteristic; AUC, area under the curve; CI, confidence interval; VPP, positive predictive value; IARS, International Anaesthesia Research Society; BJA, British Journal of Anaesthesia
