# Supplementary material for: Useful Ultrasonographic Parameters to Predict Difficult Laryngoscopy and Difficult Tracheal Intubation—A Systematic Review and Meta-Analysis
Source: Front Med (Lausanne). 2021 May 28;8:671658. doi: 10.3389/fmed.2021.671658 (PMC8193063; doi:10.3389/fmed.2021.671658)
Supplement: Supplementary file 2 [file Table_2.DOCX]

**Table 2. Relevant cut-off values with respective sensitivity (Se) and specificity (Sp), means and standard deviations of the difficult laryngoscopy group of the studies included in the meta-analysis.**

|  | Distance from skin to | | | | | Other measures | | | | | |
| --- | --- | --- | --- | --- | --- | --- | --- | --- | --- | --- | --- |
|  | **Hyoid bone** | **Epiglottis at THM level** | **Anterior commissure of Vocal Cords** | **Anterior aspect of trachea at the level of thyroid isthmus** | **Anterior aspect of trachea at the level of**  **supraesternal notch** | **HMD neutral** | **HMD extended** | **HMDR2** | **Floor of the mouth muscle volume** | **Tongue volume** | **Pre-E/mVC** |
| **Ezri 2003**  **(N = 50)** |  |  | 2.8± 0.27cm | 2.5±1.3cm | 3.3±0.43cm |  |  |  |  |  |  |
| **Komatsu 2007**  **(N=64)** |  |  | 2.04±0.3cm |  |  |  |  |  |  |  |  |
| **Adhikari 2011**  **(N = 51)** | 1.69cm  (1.19-2.19) | >2.8cm/ 3.47cm (2.88-4-07) |  |  |  |  |  |  |  |  |  |
| **Gupta 2012**  **(N = 49)** |  |  |  |  |  |  |  |  |  |  | 2.54±0.98cm |
| **Wu 2014**  **(N = 203)** | >1.28cm (Se: 85.7%; Sp: 85.1%) [1.51±0.27] | >1.78cm  (Se: 100%; Sp:66.2%) [2.39±0.34] | >1.1cm  (Se: 75%; Sp: 80.6%) [1.30±0.31] |  |  |  |  |  |  |  |  |
| **Pinto 2016**  **(N = 74)** |  | ≥2.75cm  (Se: 64.7%; Sp:77.1%) [2.825±0.443] |  |  |  |  |  |  |  |  |  |
| **Andrus 2016**  **(N = 199)** |  |  |  |  |  | 3.99±0.56cm | 4.28±0.64cm | 1.07±0.08cm | 20.10±5.39cm^3^ | 121.7±27.1cm^3^ |  |
| **Reddy 2016**  **(N = 100)** | 0.38±0.16cm |  | >0.23cm  (Se: 85.7%; Sp:57%) [0.35±0.18] |  |  |  |  |  |  |  | 1.29±0.44cm |
| **Wojtczak 2012**  **(N = 12)** |  |  |  |  |  | 5.13±0.53cm | 5.26±0.58cm | 1.02±0.01cm | 34.8±11cm^3^ | 137±29cm^3^ |  |
| **Rana 2018**  **(N = 120)** |  |  |  |  |  |  |  | ≤1.085cm  (Se:75%, Sp:85.3%)  [1.06±0.26] |  |  | >1.77cm  (Se:82%;  Sp:80%)  [1.987±0.26] |
| **Petrisor 2018**  **(N = 25)** |  | ≤1.38cm  (Se: 75%, Sp:75%) [1.575±3.073cm] |  |  |  | ≤4.47cm (Se:100%, Sp:42.86%) [4.04±0.1cm] | ≤5.5 cm (Se:100%, Sp:71.4%) [4.9±0.22cm] | ≤1.23cm  (Se: 100%, Sp:90.5%)  [1.21±0.0005] |  |  |  |
| **Alessandri 2019 (N = 194)** | 1.08±0.41cm | 0.91±0.28cm | 0.81±0.20cm | 0.86±0.31cm | 1.24±0.37cm |  |  |  |  |  |  |

**Abbreviations**: THM , thyrohyoid membrane; VC, vocal cords; HMD, hyomental distance (in neutral or extended position); HMDR2, HMD in extended position to neutral position ratio; Pre-E/mVC, pre-epiglottic distance/distance from epiglottis to the mid-point between anterior and posterior vocal cords
