# Supplementary figures and images for: Useful Ultrasonographic Parameters to Predict Difficult Laryngoscopy and Difficult Tracheal Intubation—A Systematic Review and Meta-Analysis
Source: Front Med (Lausanne). 2021 May 28;8:671658. doi: 10.3389/fmed.2021.671658 (PMC8193063; doi:10.3389/fmed.2021.671658)

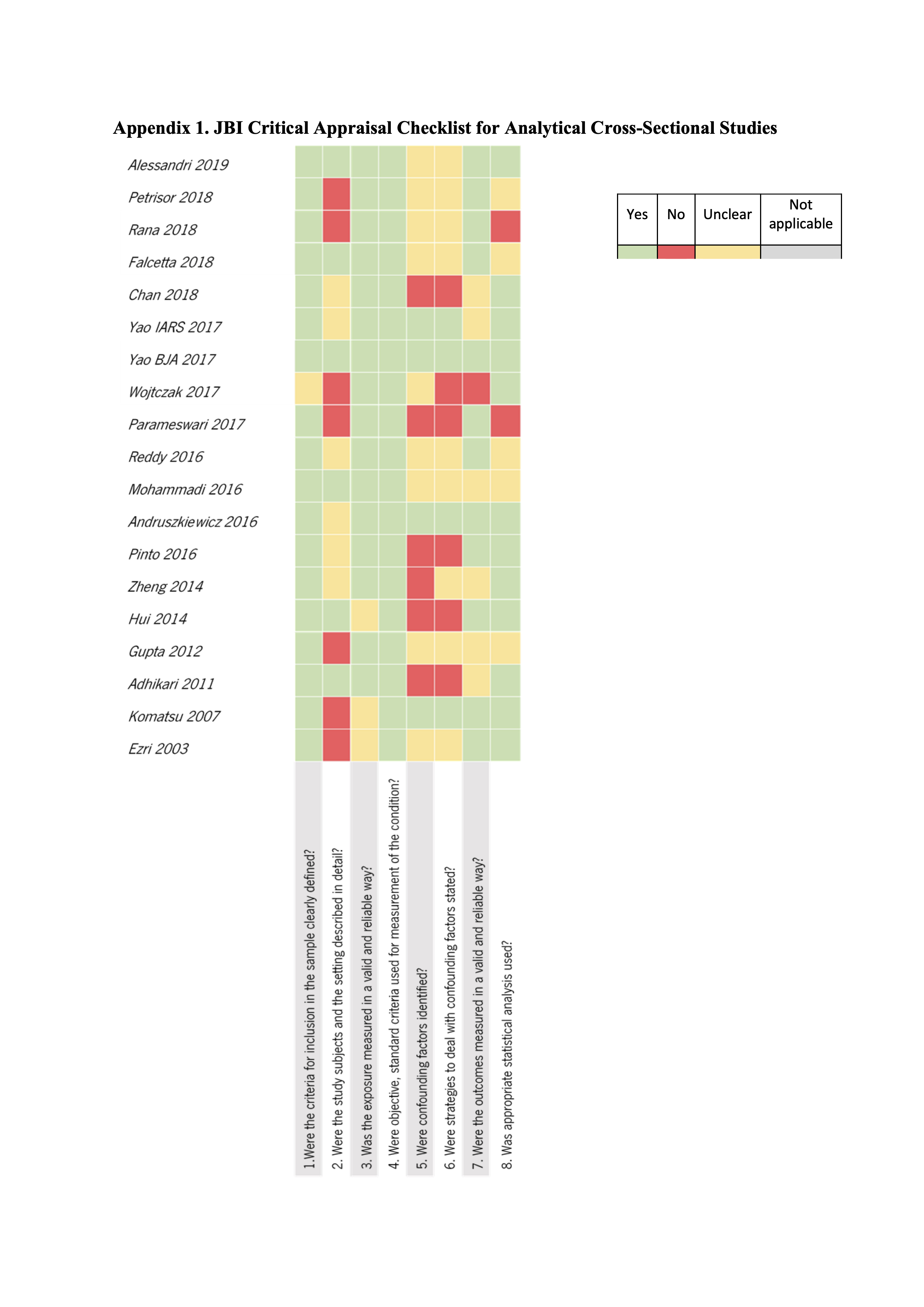

Supplement: Supplementary file 3 [file Image_1.JPEG]
